# Fetal and Neonatal Immune Response to Congenital Cytomegalovirus (cCMV) Infection: A Systematically Conducted Scoping Review

**DOI:** 10.3390/v18020242

**Published:** 2026-02-14

**Authors:** Chrysanthi-Eleni Loizou, Antonios Gkantaras, Sofia Karagiannidou, Garyfallia Syridou, Despoina Gkentzi, Vassiliki Papaevangelou, Evangelia Farmaki

**Affiliations:** 1 Third Department of Pediatrics, School of Medicine, National and Kapodistrian University of Athens, University General Hospital “Attikon”, 124 62 Athens, Greece; sofikar@med.uoa.gr (S.K.); gsyridou@med.uoa.gr (G.S.); vpapaev@med.uoa.gr (V.P.); 2 First Department of Pediatrics, Pediatric Immunology and Rhumatology Referral Center, Aristotle University of Thessaloniki, “Hippokration” General Hospital, 546 42 Thessaloniki, Greece; agkantar@auth.gr (A.G.); farmakg@auth.gr (E.F.); 3 Department of Pediatrics, Patras Medical School, University General Hospital of Patras, 265 04 Rio, Greece; gkentzid@upatras.gr

**Keywords:** congenital Cytomegalovirus, immune response, T cells, NK cells, cytokines, chemokines, transcriptomic signatures, T cell exhaustion

## Abstract

Congenital Cytomegalovirus (cCMV) infection is associated with numerous long-term sequelae. This scoping review consolidates existing evidence on fetal and neonatal immune response to cCMV and their potential relevance to clinical outcomes. A systematic search was conducted in the PubMed database. Observational studies were eligible when full text was available in English and data for immune response (innate, humoral, cellular) and/or immune-related biomarkers (cytokines and molecular markers) were provided. Thirty-four studies were included. CMV-infected fetuses mount robust γδ and CD8^+^ T-cell responses from the second trimester of pregnancy, with the transcriptomic and cytokine profile of their amniotic fluid revealing upregulation of IFN-γ-inducible genes and cytokines. cCMV-infected neonates mount oligoclonal γδ T-cell responses and functional NK and CD8^+^ T-cell responses, although data on the latter’s association with symptoms at birth are contradictory. Conversely, CD4^+^ T-cell responses are impaired, irrespective of symptoms. T cell exhaustion is an emerging finding with unclear implications on long-term outcome. Despite shared transcriptomic profiles between symptomatic and asymptomatic neonates, a 16-gene classifier biosignature has been identified for late-onset sensorineural hearing loss. In conclusion, immune response to cCMV is characterized by a Th1 signature, with T cell exhaustion being an emerging finding warranting further investigation.

## 1. Introduction

Congenital Cytomegalovirus (cCMV) infection is the most prevalent congenital infection and a leading cause of non-genetic Sensorineural Hearing Loss (SNHL) and Neurodevelopmental Impairment (NDI) [1,2,3,4]. Symptomatic newborns (10%) are at higher risk of sequelae, while 15–20% of those who are asymptomatic at birth may also have late-onset manifestations of cCMV, most frequently SNHL [2,5].

Currently, available prognostic markers associated with the development of long-term impairment (LTI) include maternal CMV infection during the first trimester of pregnancy, symptomatic disease at birth, and abnormal findings in neonatal Magnetic Resonance Imaging [6,7,8,9]. However, the presence of these markers does not consistently predict an unfavorable outcome, pointing to additional factors influencing the outcome of cCMV infection. Among these, emerging evidence implicates fetal and neonatal immune responses, with their delicate balance potentially serving as a key determinant. While robust immune responses may be protective by lowering viral load, they can also lead to immune-mediated pathology and promote an unfavorable outcome. Current evidence is scarce and heterogeneous, still lacking clinical applications [10].

The scope of this review is to consolidate evidence from published literature regarding the response of the fetal and neonatal immune system to cCMV. Even though a number of literature reviews on the topic already exist, most are narrative, while the existing systematic reviews do not efficiently explore how fetal and neonatal immune responses may affect the outcome of cCMV infection [10,11,12,13]. To our knowledge, this is the first systematically conducted review presenting published literature data on fetal and neonatal immune response to cCMV infection, aiming at addressing potential gaps, implications for clinical practice, and future research opportunities.

## 2. Materials and Methods

This study was conducted in accordance with the recommendations of the Preferred Reporting Items for Systematic Reviews and Meta-analyses Extension for Scoping Reviews (PRISMA-ScR) statement [14]. The scoping review protocol is not registered.

### 2.1. Search Strategy

A systematic search was conducted by two researchers (C.-E.L. and A.G.). Eligible articles were identified by a search of the PubMed bibliographic database for the period from 1 January 2000 up to 31 October 2025. The date restriction was applied to ensure inclusion of contemporary studies, employing more accurate methods for cCMV diagnosis and immune profiling, conforming to current clinical and scientific standards.

The search strategy included the following keywords and MeSH terms: (“Cytomegalovirus Infections”[Mesh] OR “Cytomegalovirus Infections/congenital”[Mesh] OR “cCMV” OR (“congenital” AND (“cytomegalovirus” OR “CMV”)) OR “Cytomegalovirus Infections/immunology”[Mesh]) AND (“Immunity/immunology”[Mesh] OR “Adaptive Immunity”[Mesh] OR “Immunity, Cellular”[Mesh] OR “Antigen Presentation”[Mesh] OR “Lymphocyte Activation”[Mesh] OR “Immunity, Humoral”[Mesh] OR “Immunity, Innate”[Mesh] OR “Immunity, Maternally Acquired”[Mesh] OR “B-Lymphocytes”[Mesh] OR “T-Lymphocytes”[Mesh] OR “Receptors, Antigen, B-Cell”[Mesh] OR “Receptors, Antigen, T-Cell”[Mesh] OR “Receptors, Antigen, T-Cell, gamma-delta”[Mesh] OR “Receptors, Antigen, T-Cell, alpha-beta”[Mesh] OR “Killer Cells, Natural”[Mesh] OR “Monocytes”[Mesh] OR “Neutrophils”[Mesh] OR “Antigen-Presenting Cells”[Mesh] OR “Chemokines”[Mesh] OR “Cytokines”[Mesh] OR “Interferons”[Mesh] OR “Biomarkers”[Mesh] OR “Biosignature*” OR (“Immun*” AND “pathway*”) OR (“Gene Expression Profiling”[Mesh] AND “immun* response*”)) AND((“Fetus”[Mesh] OR “fetus*” OR “fetal”) OR (“Infant, Newborn”[Mesh] OR “neonat*” OR “newborn*” OR “infan*”) OR “antenatal” OR “perinatal”). Moreover, cited references from selected articles were manually screened to identify additional studies that were not retrieved in the initial search. Conference abstracts were not searched because they do not contain sufficient data for quality assessment.

### 2.2. Study Selection Criteria

Following the literature search, identified studies were checked to exclude duplicates. The remaining articles were independently screened by 2 researchers (C.-E.L. and A.G.) to identify studies that met the predetermined inclusion criteria. The selection process was performed in 2 steps. In the first step, titles and abstracts were evaluated for eligibility against the predetermined criteria. In the second step, the full-text articles were assessed when the information provided in titles/abstracts was insufficient to decide on inclusion or exclusion. Any disagreements between the 2 researchers were resolved through discussion with a third researcher (G.S.).

The study eligibility criteria were selected by applying the PICOS (participants, interventions, comparators, outcomes, and study design) question format:

- Participants: Studies referring to human fetuses and neonates. Studies referring to amniotic fluid (AF) reflecting fetal immune responses were also eligible for inclusion.
- Interventions and Comparators: Studies including both cCMV infection and non-cCMV infection groups; control groups consisted of CMV-negative fetuses and CMV-negative newborns. Additionally, studies comparing cCMV-infected infants with varying disease severity and LTI were eligible.
- Outcomes: Studies providing data on at least one of the following factors: immune response (innate, humoral, cellular) or immune-related markers (cytokines, chemokines, other immune-related proteins and molecular markers, such as immune gene expression profiles and Single-Nucleotide Polymorphisms (SNPs)).
- Study design: Observational studies (case–control, cross-sectional, and cohort studies).

Articles not containing separate data for cCMV infection were excluded, as were studies conducted in animal models. Regarding AF studies, those reflecting placental or maternal responses or studies in which it was unclear whether maternal or fetal immune responses were reflected were excluded. Additionally, studies with a control group consisting exclusively of adults or older children were excluded due to the inherent differences in the immune system between neonates and other age groups (e.g., different CD4/CD8 and B-/T-cell ratios, cytokine production, higher Treg populations, lower memory T cells), which could confound interpretations unless comparisons were made to age-matched controls [15,16]. Finally, only studies available in full text in English were included.

### 2.3. Data Extraction

Data extraction was performed by one researcher (C.-E.L.), and the information was recorded in Microsoft Word tables (Microsoft, Redmond, WA, USA). The following data were noted: first author, year of publication, country, study design, number and diagnosis method of cCMV-infected participants, number and definition of control participants, matching criteria (if applicable), methods, and outcomes. Furthermore, results and limitations of the studies regarding fetal and neonatal immune response were recorded (Tables S1–S3).

### 2.4. Quality Assessment

Study quality was evaluated by two researchers (C.-E.L. and S.K.) using the Joanna Briggs Institute (JBI) Critical Appraisal tool [17].

## 3. Results

### 3.1. Study Selection and Characteristics

**Figure 1:**
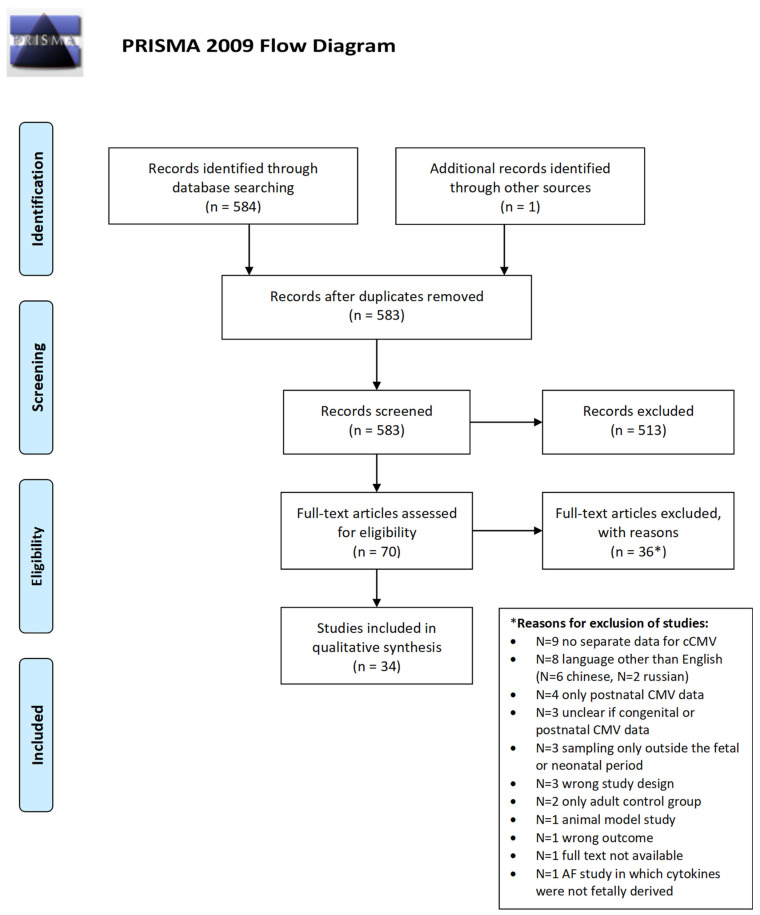
PRISMA flow chart.

Of the 583 papers identified in the literature search, 34 were included in the systematic review [18,19,20,21,22,23,24,25,26,27,28,29,30,31,32,33,34,35,36,37,38,39,40,41,42,43,44,45,46,47,48,49,50,51] (Figure 1, flow chart).

The main characteristics of the included studies are summarized in Table S1. Three studies were prospective cohorts [19,20,22], twenty-five were case–control studies [18,21,23,24,27,28,30,31,32,33,34,35,37,38,40,41,42,43,45,46,47,48,49,50,51], four were case–control studies with a nested prospective cohort [25,26,36,39], one was a prospective cohort with a nested case–control study [44], and one was a case–control study with a cross-sectional part [29]. Six studies were conducted on fetuses [18,25,28,37,39,48], twenty-four on neonates [19,20,21,22,23,26,27,29,30,31,32,33,34,35,36,38,41,42,43,44,45,46,49,51], while four enrolled both fetal and neonatal participants [24,40,47,50].

In most studies, cCMV infection was diagnosed either antenatally, through a positive CMV-DNA PCR in amniotic fluid (n = 11), and/or during the neonatal period in accordance with the 2017 European Expert consensus [52]: positive CMV-DNA PCR in urine (n = 13), or positive saliva CMV-DNA PCR confirmed by urine PCR (n = 2) obtained within 21 days of birth, or positive CMV-DNA PCR of stored Dried Blood Spots (DBS) (n = 3). Nineteen studies employed additional methods for cCMV diagnosis for some or all of their participants, including isolation of the virus in urine culture [20,25,26,27,29,36,39,47], positive CMV-DNA PCR in cord or peripheral blood [21,23,26,27,32,38,43,46,50,51], positive Immediate-Early (IE) Viral Antigen test [20,33], positive CMV-specific IgM [45,49], and isolation of CMV on Human Fetal Lung Fibroblast Cells (MRC5) monolayers [24].

In the case–control studies, non-cCMV infants, in whom cCMV infection was ruled out with a negative CMV-DNA PCR in urine or serology, were used as a control group. Furthermore, in fifteen studies, analyses were conducted based on:

(a) Prenatal findings or symptoms at birth (defined as at least one cCMV-related finding in physical examination, laboratory testing, neuroimaging, chorioretinitis, or hearing loss) [18,25,33,35,36,39,40,41,44,50,51];
(b) Severity of prenatal findings or symptoms at birth: severely symptomatic (defined as severe brain abnormalities/neurological impairment or stillbirth/terminated fetuses with severe brain lesions confirmed postmortem) versus symptomatic or asymptomatic according to the definitions provided above [18,48,49];
(c) LTI irrespective of symptoms at birth [35,41,42,44];
(d) Birth or delayed-onset SNHL, irrespective of other symptoms [31,35,36];
(e) Isolated SNHL versus severely symptomatic or asymptomatic according to previously provided definitions [48].

Studies investigating several aspects of immune response were included in this review, including cellular components of innate (γδ T cells, Natural Killer (NK) cells, Dendritic Cells (DCs)) and adaptive immunity (CD4^+^ and CD8^+^ T cells), but also soluble components such as cytokines and chemokines, as well as immune-related genes and their associated transcripts. It must be highlighted that the conducted search revealed an imbalance between studies investigating cellular and humoral immune response to cCMV infection, with only three studies examining the neonatal B cell response to cCMV, either directly or indirectly [25,40,41].

Regarding the methodological quality of the studies included, cohort studies displayed the lowest risk of bias, with variable degrees of bias attributed to case–control studies. Importantly, the exposure status (cCMV infection or lack thereof) was assessed using reliable diagnostic methods in all studies included, achieving one of our goals set by including more contemporary studies. The main limitations of the included studies, that could have introduced bias, included inadequate identification of potential confounders and/or strategies to deal with them (Tables S4–S6).

### 3.2. Synthesis of Results

#### 3.2.1. Fetal Immune Response

Nine studies investigated fetal immune response to cCMV (Table 1) [18,24,25,28,37,39,40,47,48].

Despite variability in gestational age across studies, cases and controls within each study were either matched or did not differ significantly according to a relevant statistical test. Three of these studies investigated the cytokine profile, immune gene expression, and immune protein secretion in the AF at gestational ages ranging from 18 to 25 weeks, using multiplex bead-based assays, RNA sequencing, and mass spectrometry proteomics, respectively [18,28,48].

Cytokine analysis of AF and Extracellular Vesicles (EVs) compared fetuses with CMV infection versus CMV-negative healthy fetuses, symptomatic versus asymptomatic cCMV fetuses, and fetuses with mild versus severe symptomatic cCMV infection. Fetal CMV infection was associated with an increase in six cytokines (soluble IP-10/CXCL10, IL-18, ITAC/CXCL11, TRAIL, surface IP-10, and internal IP-10). Symptomatic cCMV infection was related to the elevation of twelve cytokines, five of which were also increased in the broader cCMV infection group (soluble IP-10/CXCL10, IL-18, TRAIL, surface IP-10, and internal IP-10). Finally, six cytokines were related to severe symptomatic infection (soluble IL-18, soluble TRAIL, CRP, surface TRAIL, internal MIG, and internal RANTES) [18].

The second study applied RNA sequencing in the AF, unraveling upregulation of 190 genes and downregulation of 32 genes in CMV-infected fetuses compared to CMV-negative ones. The upregulated genes were mainly involved in the innate immune response (N = 70), IFN signaling (N = 28), and immune response to viruses (N = 38). In contrast, 8 out of 32 downregulated genes were related to neurodevelopment. After excluding fetuses with ultrasound abnormalities at recruitment, the number of upregulated genes decreased (N = 63), though the categories of these genes remained unchanged. Only one gene related to neurodevelopment, periaxin, remained downregulated after this adjustment [28].

The third study applied mass spectrometry proteomics to analyze AF samples from severely symptomatic cCMV-infected fetuses, asymptomatic cCMV-infected fetuses, and CMV-negative fetuses and detect differentially secreted proteins between groups. Overall, 59 proteins were differentially secreted between cCMV-infected and CMV-uninfected fetuses, whereas 29 proteins were differentially secreted between severely symptomatic and asymptomatic cCMV-infected fetuses. Immunomodulatory proteins chemerin and galectin-3–binding protein (Gal-3BP) showed consistently higher levels in cCMV-infected fetuses and in those with severe disease. Lower levels of both proteins were observed in fetuses with isolated congenital or delayed-onset SNHL compared with severely symptomatic cases, while no differences were detected between isolated SNHL and asymptomatic cCMV infection [48].

Two studies investigated β2 microglobulin levels in the AF and fetal blood of CMV-infected fetuses and healthy controls, with higher levels correlating with cCMV infection in both sample types [25,39]. Additionally, among CMV-infected fetuses, higher β2 microglobulin levels were related to symptomatic status, with an AUC of 0.98 in fetal blood [25]. The AF study also investigated soluble HLA-G (sHLA-G) levels, demonstrating higher levels and sHLA-G index, but also the presence of free heavy chains in CMV-infected fetuses compared to healthy ones, as well as symptomatic fetuses compared to asymptomatic ones [39].

Four studies explored cellular responses in cord blood; one explored γδ T cells, while three addressed CD8^+^ T cell responses of CMV-infected fetuses compared to uninfected ones during mid- and late gestation [24,47]. In general, increased proportions of total T lymphocytes were present in CMV-infected fetuses compared to uninfected ones, with the highest proportions detected in symptomatic fetuses [25]. Robust responses were detected in the second trimester of pregnancy in both γδ and CD8^+^ T-cells (at 21- and 22-weeks gestational age, respectively) [24,47].

The γδ T-cells from CMV-infected fetuses displayed a late differentiation phenotype (CD27-CD28-) and increased expression of NK receptor and cytotoxic mediator genes (perforin, granzyme A) compared to CMV-negative fetuses. Moreover, specific CDR3δ1 and CDR3γ8 sequences, corresponding to a public T-Cell Receptor (TCR) enriched in cCMV-infected newborns, were already present in CMV-infected fetuses at this early stage of gestation [47].

CD8^+^ T-cells in CMV-infected fetuses preferentially expanded over CD4^+^ T-cells and displayed a switch towards more mature phenotypes, including memory (CD45RA^−^ or CD45RO^+^), activated (HLA-DR^+^), and terminally differentiated (CD28^−^) cells, compared to CMV-uninfected fetuses [24,37]. Also, T-cells of CMV-infected fetuses were able to secrete Interferon-γ (IFN-γ) following mitogen stimulation (Ca^+^/PMA); however, their response to CMV antigen was significantly weaker and comparable to the one elicited in CMV-negative fetuses [24].

Finally, two studies explored humoral CMV-specific responses in fetuses, with contradictory results; in the first study, CMV-specific IgM levels did not differ between symptomatic and asymptomatic CMV-infected fetuses [40]. However, a subsequent study detected higher CMV-specific IgM levels in symptomatic fetuses that could accurately distinguish them from asymptomatic ones (AUC 0.95) [25].

#### 3.2.2. Neonatal Immune Response

Twenty-seven studies investigated neonatal immune response to cCMV (Table 2, Table 3 and Table 4) [19,20,21,22,23,24,26,27,29,30,31,32,33,34,35,36,38,40,41,42,44,45,46,47,49,50,51]. Seven studies explored innate immunity, twelve studies investigated adaptive immunity, and twelve studies focused on immune markers.

##### Innate Immunity

Compared to healthy controls, cCMV-infected neonates generally exhibit higher absolute numbers and percentages of total γδ T cells. A switch towards more mature phenotypes is observed; γδ T-cells are activated (HLA-DR^+^), differentiated (CD27^−^CD28^−^), and express higher amounts of NK receptor genes (Killer Immunoglobulin Receptor (KIR), CD94/NKG2A/NKG2C, natural cytotoxicity receptor family). They also express elevated levels of cytotoxic mediator (mainly granzyme B and H, but also granzyme A, granzyme M, perforin, TRAIL, granulysin, and FasL) and chemokine/chemokine receptor (MIP-1α/CCL3, MIP-1β/CCL4, RANTES/CCL5, CCR5 ligands, CCR5, and CX3CR1) genes. Importantly, a CMV-specific Vγ8Vδ1 public TCR was discovered in cCMV-infected newborns, which, upon CMV stimulation, produced IFN-γ and contributed to the restriction of viral replication and killing of CMV-infected cells [47].

Three studies investigated NK cell responses; no significant differences in NK cell frequencies between cCMV-infected neonates and healthy controls were detected [46]. However, more mature NK cell subsets predominated in cCMV-infected neonates, with a significantly higher frequency of CD56^neg^ and CD56^dim^ subsets, irrespective of symptoms. These subsets expressed activation, differentiation, and memory markers (CD57^+^, Programmed cell Death protein-1 (PD-1^+^), KIRs). The expression pattern of NKG2 receptors, however, seems more complex. While the NKG2C activating receptor was upregulated in all cCMV-infected neonates in two studies, regardless of symptoms, in the third study, this finding was limited to symptomatic ones. Downregulation of the NKG2A inhibitory receptor was consistent across all three studies, while the activating NKG2D receptor was downregulated in one study [38,43,46]. Functionally, NK cells from cCMV-infected neonates showed enhanced cytotoxic potential, characterized by CD7 downregulation, enrichment in cytotoxic mediators (mainly granzyme B and perforin), and frequent co-expression of multiple cytotoxic molecules [46]. Degranulating NK cells (CD107a^+^) also expressed high levels of NKp46 activating receptor [38].

DCs have only been studied in a transcriptomic in vitro study. In this study, CMV-infected cord blood-derived DCs upregulated several cytokine genes (IL-1α, IL-1β, IL-7, IL-15), chemokine genes (CCL2/MCP-1, CXCL4/MIP-1β, CCL5/RANTES, CCL7/MCP-3, CCL8/MCP-2, CCL20/MIP-3α, CXCL8/IL-8, CXCL9/MIG, CXCL10/IP-10, CXCL11/ITAC), IFN genes (initially IFNA1 and IFNB1, followed at 16 h post stimulation by IFNA2, IFNA4, IFNA5, IFNA7, IFNA8, IFNA10, IFNA13, IFNA14, IFNA16, IFNA21 and IFNW1) and immune receptor genes (TLR7). In contrast, genes related to lipid metabolism and immunoregulatory G-Protein Coupled Receptors (ADORA3, ARRB1, C5AR1, CXCR4, GPR34, GPR183, GRK3, and RGS18) were downregulated [23].

Regarding the complement system, no association was found between Mannose-Binding Lectin 2 (MBL2) deficiency or polymorphisms and cCMV status (infected and uninfected newborns). The potential impact of these factors on symptomatic or asymptomatic disease at birth was not investigated [45]. Finally, higher levels of C3 have been related to symptomatic cCMV disease, including isolated SNHL, though not statistically significant [51].

##### Adaptive Immunity

Eight studies investigated CD4^+^ T cell responses to cCMV infection (Table 3) [20,24,27,29,32,35,43,44]. In cCMV-infected neonates, CD4^+^ T cell responses were generally weaker than CD8^+^ T cell responses in terms of IFN-γ-producing cells following CMV antigen stimulation, with their levels frequently being undetectable. Low CD4^+^ T cell responses generally persisted during the first year of life, with a gradual increase noted over the first 3 years of life [20,29,32,35]. Findings regarding phenotype markers are conflicting; CMV-specific CD4^+^ T-cells were characterized by a switch towards memory phenotype (CD45RA^−^ or CD45RO^+^) in one study [24], but subsequent studies failed to replicate this finding [27,35,43]. CMV-specific CD4^+^ T cells are terminally differentiated (CD28^−^), and while one study detected increased activated and exhausted (HLA-DR^+^ CD57^+^ PD-1^+^) CD4^+^ T cell populations, another one did not demonstrate such a difference [29,35,43]. No statistically significant differences in total CD4^+^ T cell count or CMV-specific CD4^+^ T-cell responses were found between symptomatic and asymptomatic cCMV-infected newborns or between those who presented LTI and those who did not [44].

Eleven studies evaluated CD8^+^ T cell responses to cCMV infection (Table 3) [19,20,24,26,27,29,32,34,35,43,44]. In cCMV-infected neonates, an oligoclonal expansion of CD8^+^ over CD4^+^ T-cells was observed. Although naïve phenotypes predominated, when compared to CMV-negative neonates, a switch towards memory (CD45RA^−^ or CD45RO^+^), activated (HLA-DR^+^), and terminally differentiated (CD28^−^) phenotypes was observed [24,29,34,35]. In one study, no differences in the total frequencies of memory CD8^+^ T cells were observed between groups, despite a predominance of effector memory RA CD8^+^ T cells (CD45RA^+^ CCR7^−^) in cCMV-infected neonates [27]. Notably, lower proportions of effector memory and effector memory expressing RA subsets have been detected in cCMV infants with developmental delay [35]. CD8^+^ T-cells in cCMV-infected neonates are able to mount functional responses; they are cytotoxic (high perforin levels), they produce cytokines (MIP-1β/CCL4, TNF-α), and they secrete IFN-γ in response to CMV [24,32,34]. Interestingly, NK-like CD8^+^ T cell populations were detected in one study, which were characterized by the expression of NKG2C, NKG2A, KIRs, and FcγRIII receptors, as well as cytotoxic content. These cells mediated Antibody-Dependent Cellular Cytotoxicity (ADCC) by degranulating cytotoxic mediators and secreting IFN-γ in an FcγRIII-dependent manner [43]. A gradual increase in CD8^+^ T cell responses is observed over the first 3 years of life and has been associated with the decrease in urine viral load [20,26,35]. Moreover, the earlier development of a detectable CD8^+^ T cell response appeared to correlate with an asymptomatic status at birth and a lower risk of LTI, as observed over a median follow-up of 30 months [19]. However, another study did not confirm this association and found no relationship between total CD8^+^ T-cell counts or CMV-specific CD8^+^ responses and LTI at a median follow-up of 25.3 months, although a lower total T-lymphocyte count was significantly associated with LTI [44]. Interestingly, an increased expression of PD-1, a marker of T cell exhaustion, has been observed in both CD4^+^ and CD8^+^ T cell populations in cCMV newborns [29,35,43]. Its persistent expression in CD8^+^ T cells was linked to progressive SNHL in one study, while its combined expression with activation (HLA-DR^+^) and terminal differentiation (CD57^+^) markers has been linked to asymptomatic status at birth and normal neurodevelopment [35].

Finally, one study explored CMV-specific IgM responses, linking higher responses to symptomatic cCMV at birth [40].

##### Immune-Related Markers

Of the twelve studies investigating potential immune-related biomarkers in cCMV infection, three focused on cytokines and chemokines [21,33,49], one on immune-related proteins [51], one on T-Cell Receptor Excision Circles (TRECs) and Kappa-deleting Recombination Excision Circles (KRECs) [41], while the remaining seven involved genomic approaches and gene expression signatures (Table 4) [22,23,30,31,36,42,50].

Regarding cytokines, increased levels of IL-33 and soluble Suppression of Tumorigenicity 2 (sST2) in the unstimulated serum of cCMV-infected infants compared to CMV-negative controls were noted [21]. Another study using unstimulated serum samples found that cCMV-infected infants had higher levels of β-defensin 8 and macrophage-derived chemokine compared to non-CMV-infected infants. According to the same study, hepatitis in cCMV-infected neonates was associated with increased levels of circulating PF-4/CXCL4 and IL-25, whereas elevated levels of β-defensin 31 were associated with its absence [33]. Lastly, analysis of cerebrospinal fluid (CSF) revealed increased Acrp30 and MMP-3 levels and lower IL-1α levels in symptomatic cCMV neonates compared with CMV-negative febrile neonates; increased MMP-3 levels persisted when cCMV-infected neonates with severe abnormalities were compared to those with mild ones [49].

One study used proteomics to detect differentially excreted proteins among infants with variable disease severity and the presence or absence of neuroimaging findings. 80 proteins were differentially secreted between infants with symptoms or isolated SNHL and asymptomatic ones, 65 of them upregulated and belonging to pathways related to complement and coagulation cascade, platelet degranulation, and inflammatory response. The protein displaying the highest fold change (168.24) was Interferon-induced guanylate-binding protein 1, while several components of the complement pathway were upregulated, but only C3 levels were validated using ELISA. In the same cohort, infants with neuroimaging abnormalities differentially expressed 31 proteins, with 30 of them upregulated and mostly belonging to pathways related to the regulation of insulin-like growth factor (IGF) transport and uptake by IGF proteins and response to wounding. The top enriched protein in these infants is one of unknown function, encoded by the KIAA1109 gene, while the single downregulated protein is CYP39A1, a monooxygenase of CYP450 [51].

One study conducted in DBS samples showed that cCMV-infected neonates had statistically significantly lower TREC counts than healthy controls. Also, a specific TREC rearrangement (Vδ1Jδ1) was detected in more γδ T cells in cCMV-infected neonates and was related to higher viral loads. On the other hand, KREC numbers and percentages did not differ between cCMV-infected neonates and healthy controls. However, a higher percentage and number of KRECs were associated with higher viral loads and a specific rearrangement (cjintronRSS-Kde). There were no statistically significant correlations between TREC and KREC parameters and symptoms at birth. However, a statistically significantly lower percentage and number of KRECs were detected in the LTI-free cCMV-infected infants [41].

Finally, seven studies explored genes potentially influencing the outcome of cCMV infection. Four studies using whole blood samples focused on SNPs in immune-related genes and their relation to cCMV [22,30,31,50]. Two SNPs in the IL1B gene have been studied, with both of them linked to increased risk of cCMV infection but opposite findings in terms of symptoms; the first SNP was related to symptomatic cCMV disease, while the second one was related to reduced risk of ventriculomegaly on neuroimaging and reduced risk of splenomegaly [22,30,31,50]. An SNP in the IL28 gene was linked to increased risk of thrombocytopenia, but also cystic lesions and ventriculomegaly on neuroimaging [22,30]. Finally, four SNPs in genes encoding IL-12, TLR4 [30], and MCP-1/CCL2 [31] were linked to three distinct signs and symptoms: reduced risk of prematurity and increased risk of hepatitis [30] and SNHL at birth, respectively [31]. CCL2 rs1024611 polymorphism was related to increased risk of SNHL at birth in one study [31], but the other one failed to replicate this finding [30].

Three studies applied transcriptomics on blood or DBS from infants with cCMV to uncover Differentially Expressed Genes (DEGs) between cCMV-infected and CMV-negative neonates, as well as among cCMV-infected neonates with different outcomes. One study comparing a symptomatic cCMV neonate to 13 non-cCMV neonates with bacterial sepsis revealed increased expression of NK and DC markers, as well as upregulation of cell cycle and IFN-induced gene networks [23]. Another study identified biosignatures distinguishing cCMV-infected from CMV-negative neonates, although distinction between symptomatic and asymptomatic neonates based solely on biosignatures was not feasible due to substantial overlap between groups [36]. Also, another study correlated higher CMV viral loads with increased expression of genes related to T cell differentiation (CD57, T-bet), effector (IFN-γ, granzyme), and inhibitory markers (PD-1, Lymphocyte-Activation Gene 3 (LAG3)), consistent with T cell exhaustion [42]. Finally, while no DEGs or pathways were linked to LTI, a 16-gene classifier signature was identified, predicting which asymptomatic infants would develop late-onset SNHL (97% AUC). Two of the genes were non-protein coding (LOC286135, LOC645431), whereas twelve genes were involved in various processes, including cell cycle and homeostasis (AK3L1, RAB9B, RABGAP1), chromatin remodeling (MYST2/KAT7, PAXIP1, JMJD2A/KDM4A, LEO1), lipid synthesis and use (MPDU1), innate immune response (MYST2/KAT7, MATR3, CLEC4G), immune regulation (CD40), and neurodevelopment (ARHGEF9). Lastly, one gene encodes a protein of unknown function (GLCCI1), while another one belongs to the oxidoreductase activity category (C10orf59/RNLS) [36].

## 4. Discussion

**Figure 2:**
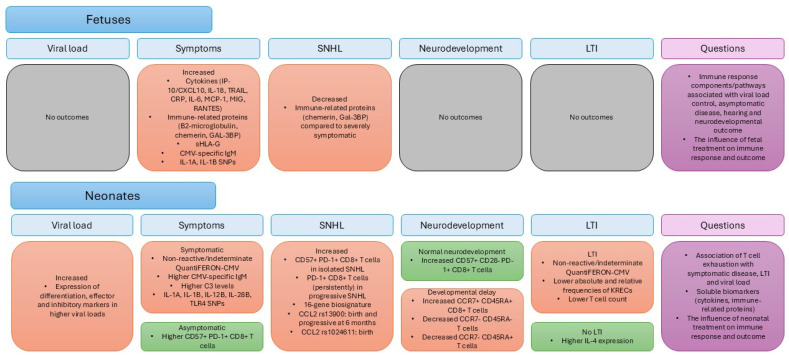
Current knowledge on immune biomarkers in relation to viral load, clinical outcomes, and future directions. Color index: Orange: related to unfavorable clinical or laboratory characteristics or outcome. Green: related to lack of symptoms or LTI. Grey: outcome not investigated.

cCMV infection is the most common congenital infection worldwide and an important cause of childhood morbidity [1,2,3]. Despite progress in treating symptomatic newborns and identifying LTI, treatment of mildly symptomatic newborns is still controversial [53,54,55,56,57]. Additionally, asymptomatic newborns are not currently treated, mainly due to the absence of reliable predictive biomarkers indicating which children are at risk of LTI and thus may benefit from antiviral treatment [58]. To date, the only prognostic factors identified among cCMV-infected newborns with clinically inapparent CMV disease include maternal infection during the first trimester of pregnancy and neuroimaging findings at birth [6]. In recent years, groundbreaking research has provided insights into the complex nature of the fetal and neonatal immune system. Understanding immune pathways associated with early viral control may correlate with the outcome of mildly symptomatic and asymptomatic cCMV newborns, hinting at biomarkers that could ultimately guide clinical decision-making (Figure 2) [59].

Innate immunity represents the first line of defense against pathogens and, in the context of cCMV infection, emerges as a critical determinant influencing both viral control and disease outcome through orchestration of CMV-specific adaptive immune responses by shaping the cytokine milieu and antigen presentation. Emerging evidence suggests the significant role of Antigen-Presenting Cells (APCs) like DCs, as they are key mediators and enhancers of T cell immune responses, which appear to be pivotal in the immune response to cCMV [60]. Transcriptomic data from cord-derived DCs following in vitro inoculation with HCMV revealed a Th1 signature during cCMV infection, characterized by upregulation of IFN-related gene networks and IFN-inducible genes. In parallel, chemokines such as MIG/CXCL9, IP-10/CXCL10, and ITAC/CXCL11, which act mainly on activated T cells expressing the CXCR3 receptor, are upregulated, and their expression increases with time [23,61,62,63]. Notably, ITAC/CXCL11 expression was significantly upregulated in the AF of CMV-infected fetuses, irrespective of clinical presentation, compared to uninfected ones, suggesting a Th1-driven immune response to CMV [18]. In the same study, expression of IL-18, a cytokine secreted by APCs that activates CD8^+^ T cell cytotoxicity and IFN-γ production, was elevated in the AF from CMV-infected fetuses compared to uninfected ones and correlated with disease severity [18,64]. Indeed, fetal CD8^+^ T cells can produce IFN-γ, as demonstrated by flow cytometry, advocating for functional cytotoxicity of CD8^+^ T cells in utero and indirectly suggesting a potential role for IL-18 in this context, even though such responses may be modulated by the intrauterine environment [24]. These findings support the notion that the IFN-γ pathway is strongly activated during the early stages of fetal immune response to cCMV and may play a role in limiting viral replication.

Beyond these early Th1-skewed responses mediated by APCs, the temporal regulation of additional cytokines and chemokines highlights the complex balance between effective viral control and potential immune-mediated tissue damage in cCMV infection. In contrast to the sustained upregulation of certain cytokines, others, such as IL-1α and IL-1β, seem to be transiently upregulated and subsequently downregulated, possibly due to the concomitant upregulation of type I IFN genes [23,65]. Evidence shows that CCL2/MCP-1, a chemotactic factor attracting monocytes in inflammatory sites, follows a similar temporal pattern. Specifically, in vitro studies show that HCMV induces early expression of CCL2/MCP-1, likely to promote monocyte recruitment and viral dissemination, but suppresses it at later stages, potentially as an immune evasion mechanism [66]. Interestingly, polymorphisms associated with increased CCL2/MCP-1 expression and heightened inflammatory responses have been linked to SNHL, supporting animal model data suggesting that SNHL may result from an excessive inflammatory response rather than direct viral damage [31,67]. The contribution of inflammation is further supported by the upregulation of the CD40 gene, which has been identified as one of the 16 genes of a biosignature predicting which cCMV neonates will develop delayed-onset SNHL [36]. CD40 is a co-stimulatory molecule expressed on APCs and B cells, which, upon binding to its ligand CD40L, initiates signaling via pathways such as NF-kB and MAPK, inducing transcription of a multitude of proinflammatory genes [68]. Therefore, we could imply that the regulation of pro-inflammatory cytokines and chemokines may influence not only disease severity but also long-term outcome, even though supporting data are limited. In this context, antioxidants have been used as a potential adjunct therapy reducing inflammation associated with SNHL; however, no benefit was detected in cCMV-related SNHL [69]. Other reported alterations in the cytokine/chemokine profile of CMV-infected fetuses and neonates mostly constitute isolated findings that cannot be interpreted due to limited understanding of their role in HCMV infection (IL-33, PF-4/CXCL4, GAL-3BP) or even in viral immunity in general (chemerin, IL-25) [21,33,48,70,71,72,73,74,75].

Shaped by the APC-mediated Th1 environment and dynamic cytokine signals, fetal T cells appear to be transcriptionally and functionally programmed for cytotoxic responses during cCMV infection. In particular, T-bet, a key transcription factor for Th1 polarization, is markedly activated during cCMV infection, promoting the differentiation of T-cells into cytotoxic effector cells [76]. Several studies have demonstrated early activation and cytotoxic differentiation of both conventional and unconventional T-cell subsets in cCMV-infected fetuses [24,25,29,37,47]. Of note, increased levels of β2 microglobulin, a component of Major Histocompatibility Complex (MHC) Class I molecules, were reported in both the AF and blood of CMV-infected fetuses, suggesting increased T cell activation and turnover [77]. Furthermore, the only transcriptomic study in AF to date reported that STAT1, which regulates T-bet expression, was one of the most upregulated genes in CMV-infected fetuses [28,76].

In γδ T cells of cCMV-infected neonates, increased expression of T-bet and eomesodermin transcription factors further supports the presence of activated and differentiated cells [47]. However, the response of neonatal αβ T cells appears more complex. Increased T-bet expression, along with enhanced granzyme and IFN-γ secretion, is observed in some studies of cCMV-infected neonates [24,29,34,36,42]. However, chronic antigenic stimulation—such as that seen in chronic viral infections—may lead to T cell exhaustion, which is characterized by the upregulation of eomesodermin transcription factor and downregulation of T-bet and triggers the expression of inhibitory markers like PD-1 and LAG3 [78]. T-cell exhaustion, a well-documented feature of chronic viral infections, is an emerging finding in cCMV infection, though it has been insufficiently explored. Huygens et al. and Rovito et al. reported increased PD-1 and LAG3 expression in cCMV-infected neonates compared to CMV-negative controls, with these markers correlating with viral loads [29,42]. Similarly, Ouellette et al. also identified LAG3 among the 10 most overexpressed genes in cCMV-infected neonates compared to CMV-negative ones, regardless of symptoms [36]. Interestingly, expression of such markers has also been demonstrated in brain cells of cCMV-infected fetuses and correlated with disease severity, supporting evidence regarding the potential role of T cell exhaustion in the pathogenesis of cCMV [79].

Interpreting exhaustion markers in relation to symptoms and LTI remains challenging. The expansion of CD8^+^ T cells in utero potentially limits CMV-induced inflammation and seems to prevent the appearance of symptoms while simultaneously leading to the acquisition of an exhausted phenotype, as depicted by the upregulation of PD-1 [35]. Current literature suggests that SNHL and NDI are two conditions with distinct pathophysiology and different associated biomarkers [80]. The predominance of exhausted CD8^+^ T cells in infants with normal neurodevelopment may suggest that in utero events and responses mostly influence neurodevelopmental outcomes, while continuous antigenic stimulation during infancy may drive progressive SNHL consistent with persistently high levels of PD-1^+^ CD8^+^ T cells in affected infants. Importantly, none of these infants’ mothers had received antiviral treatment during pregnancy [35].

Remarkably, distinct populations, such as NK-like CD8^+^ T cells, have been reported to expand during chronic HCMV infection in adults, but also during cCMV infection [81]. The expansion of such subsets may reflect an attempt of the immune system to re-engage and reprogram the exhausted and terminally differentiated CD8^+^ T cells through FcγRIII receptor engagement and ADCC [43]. Although their relationship with clinical outcomes has not been explored, the association of delayed-onset SNHL with increased CD40 expression—a molecule central to ADCC—raises the question of whether detection of such subsets could serve as an indicator of LTI risk [36].

Collectively, these findings support the involvement of T cell exhaustion in cCMV pathogenesis. Nevertheless, the underlying mechanisms remain poorly understood. Factors such as maternal infection timing and treatment during pregnancy, as well as their implications and impact on long-term outcome, warrant further investigation. This emerging evidence may also help explain inconsistent IFN-γ responses and the variability in long-term outcomes observed among cCMV-infected neonates [19,44].

The observed imbalance between reported humoral and cellular immune responses in cCMV infection is potentially attributed to the fact that cellular and cytotoxic T-cell responses are mainly involved in the effective control of viral infections, such as CMV infection [82]. Although in older human subjects, humoral immunity is established early in CMV infection, its protective value is still debated [83]. In cCMV, reports suggesting a potential protective role for humoral immunity—such as higher KREC counts in infants with favorable outcomes—should be interpreted cautiously, as stronger humoral responses reflected by higher CMV-specific IgM levels have been associated with symptomatic disease [40,41].

Regarding NK cell responses to cCMV, the literature is scarce. HCMV encodes the UL40 peptide, which can be presented by the non-classical Major Histocompatibility Complex (MHC) class I molecule HLA-E to NK cell receptors such as NKG2A and NKG2C and mediate inhibition or activation of NK cells, respectively [84,85,86,87]. Viral evasion is facilitated via NKG2A binding. In cCMV-infected neonates, NK cells appear to overcome immune evasion mechanisms by downregulating NKG2A expression [38,46]. Since NKG2C has a six-times lower affinity for HLA-E than NKG2A, the downregulation of NKG2A may be critical for effective NK cell-mediated cCMV control. The upregulation of the NKG2C receptor in cCMV-infected infants is a depiction of their activated status, which, according to one study, is independent of symptoms, while another one limits it to symptomatic neonates. Of note, the definition of symptomatic infants was different in these studies; microcephaly and Small for Gestational Age (SGA) were the only symptoms taken into account in the study that linked increased NKG2C expression to symptoms at birth, which could have confounded results [38,46]. Additionally, NK cells in cCMV-infected neonates are functionally active. Even populations such as CD56^neg^ NK cells, which expand during chronic viral infections, show prominent cytotoxicity. It is worth mentioning that these functional changes could not be attributed to differences in T-bet expression between cCMV-infected neonates and CMV-negative ones, suggesting possible post-transcriptional regulation [46].

Challenging the classic notion of robust immune responses equaling better infection control and, consequently, outcome, seems appropriate in the context of a congenital infection such as cCMV, based on the aforementioned findings. Fetal and neonatal immune responses are generally Th2-skewed, acting protectively towards detrimental, excessive inflammatory responses, which could adversely affect development. Infectious hits, however, influence immune responses, which, in the context of cCMV, are Th1-skewed [12,88]. Thus, we could hypothesize that the balance between Th1- and Th2-skewed responses could be a major determinant of long-term outcome, as adequate viral control along with minimum immune-mediated damage may be achieved in this context. Interestingly, this “immune paradox” has been described in Zika virus infections, in which predominance of Th1-mediated immune responses leads to rapid viral clearance but increased immunopathology and permanent neurological damage, while predominance of Th2-mediated immune responses promotes viral persistence, which may lead to long-term complications [89].

In terms of clinical application, the administration of antiviral treatment during pregnancy and early infancy may have indirect immunological effects, primarily through reduction in viral replication and antigenic burden. Although valacyclovir, administered at high doses until the end of pregnancy once fetal infection has been established, relies on expert opinion, as evidence regarding its efficacy in preventing symptomatic infection is insufficient, it may lead to a reduction in viral load, which could theoretically attenuate excessive immune activation. Similarly, administering valganciclovir in the neonate could suppress Th1-mediated responses and reduce ongoing inflammation. However, whether antiviral treatment exerts specific immunomodulatory effects on fetal or neonatal immune polarization remains unknown and warrants further investigation.

This hypothesis also puts into perspective the role of T cell exhaustion in long-term outcomes. Could the inability of exhausted T cells to respond effectively to cCMV be a protective mechanism of the immune system, trying to counteract excessive immune response and maintain a Th1/Th2 balance? And if this is the case, keeping in mind that monoclonal antibodies targeting immune checkpoint receptors such as PD-1, which have been detected in cCMV-infected newborns, could be used to reverse this exhausted state, as demonstrated by Huygens et al., would such an approach cause more harm than good?

Furthermore, adoptive CMV-specific T-cell transfer is currently being studied in the context of cCMV infection as an immunotherapy in severely affected infants [90]. Although this approach has shown remarkable results in immunosuppressed, transplanted patients, congenitally infected CMV neonates are a population with distinct immune characteristics in whom its effect cannot be predicted [91]. While such an intervention may boost the neonatal immune response, it could also heighten Th1-mediated responses in an immune system that has been deliberately “paralyzed” to prevent further immune-mediated damage. Whether this is a safe approach to effectively controlling cCMV infection and improving long-term outcome or a potential salvage therapy for severe cases remains to be answered.

Finally, numerous attempts at developing a CMV vaccine for use in pregnant women have been undertaken in recent years, mostly focusing on preventing fetal transmission of the virus, without showing the anticipated promise thus far [92]. The complexity of CMV as a virus, with its multiple immune evasion mechanisms, as well as the elaborate and largely unknown interactions between the virus and the host at the maternal-fetal interface, make the development of a CMV vaccine particularly challenging. The notion of a therapeutic vaccine, exploiting the functional fetal immune responses as detailed in this review, seems like a distant prospect due to insufficient knowledge of the maternal and fetal immune response to cCMV at different times of gestation, as well as the difficulties associated with the design of a study exploring these mechanisms [93].

Overall, we could argue that while several immune aspects and candidate biomarkers have been explored over the years, drawing reliable conclusions remains challenging due to the heterogeneity of published studies and methodological limitations. Many findings have either not been validated in subsequent studies or constitute non-specific inflammation markers whose prognostic value for cCMV is unclear. Future research could focus on joint transcriptomic and proteomic approaches, where transcriptomic findings followed by proteomic validation might control for potential epigenetic modifications. Finally, serial, longitudinal measurements could ensure the identification of a consistently recovered biomarker with a high prognostic value. In addition, with T cell exhaustion being an emerging finding in cCMV, which may affect outcome, future studies could aim at simultaneously assessing cytokine production and exhaustion markers (e.g., PD-1, LAG3) in CMV-specific T cells of infants with various outcomes to better delineate this relationship.

### Limitations

This review has several limitations. Language restrictions could have introduced bias, since only studies whose text was available in English were evaluated. Additionally, only one database (PubMed) was searched for relevant articles, and date restrictions were introduced; thus, some eligible studies may not have been retrieved. The possibility of publication bias cannot be excluded, since conference abstracts, unpublished dissertations, and ongoing trials were not searched, while we cannot rule out the possibility of additional studies being retrieved using a search strategy incorporating different terms. Additionally, even though the exclusion of studies without an appropriate control group, such as those with an adult control group, is clinically relevant, we cannot exclude the possibility of such studies containing important data that were not identified in our review. Finally, a meta-analysis was not feasible due to the heterogeneity of the studies included and the lack of sufficient statistical data. However, given the limited available data, we believe that consolidating existing evidence provides valuable insight and will promote further research in the field.

## 5. Conclusions

Fetal and neonatal immune responses to cCMV appear to be characterized by a pronounced IFN-γ-driven signature, marked by the upregulation of chemokines, such as ITAC/CXCL11, IP-10/CXCL10, and MIG/CXCL9. This signature reflects a robust Th1 response with increased secretion of pro-inflammatory cytokines (TNF-α, IL-1α/β, IL-18) and induction of CD8^+^ cytotoxic T-cell activity. While Th1 polarization and activation of CD8^+^ cytotoxic T cells are likely to contribute to controlling the infection, the observed variability in these immune responses across different groups of infected neonates—as well as the delicate balance between immune activation and tolerance—adds complexity to disease outcomes and warrants further investigation.

Moreover, the concurrent emergence of T cell exhaustion—characterized by impaired effector function and increased inhibitory receptor expression—introduces additional complexity to the immune landscape of cCMV. Understanding the precise mechanisms that lead to T cell exhaustion, including the role of maternal infection timing, prenatal interventions, and their impact on disease progression and long-term outcomes, remains a key research priority. Clarifying these pathways could provide insight into disease progression, help identify prognostic biomarkers, and inform more targeted therapeutic approaches.

## Figures and Tables

**Table 1:** Main findings of studies related to fetal immune response to cCMV.

Author (Year)	Immune Component Studied	Results
Bourgon (2022) [18]	Cytokines	Increased expression of Th1-related cytokines in the AF of 1. CMV-infected fetuses (IP-10/CXCL10, ITAC/CXCL11, IL-18, TRAIL). 2. Symptomatic cases (IP-10/CXCL10, IL-18, TRAIL). 3. Severely symptomatic cases (IL-18, TRAIL).
Elbou Ould (2004) [24]	CD8^+^ T cells	Functional responses detected at 22 weeks GA. Preferential expansion of CD8^+^ over CD4^+^ T cells, with a switch towards more mature phenotypes (memory, activated, terminally differentiated) and secretion of IFN-γ.
Fabbri (2011) [25]	T cells, CMV-specific IgM, β2 microglobulin	Increased proportion of total lymphocytes in CMV-infected fetuses compared to healthy controls, as well as in symptomatic cCMV compared to asymptomatic ones. Increased CMV-specific IgM in symptomatic CMV fetuses compared to asymptomatic (AUC 0.95). Increased levels of β2 microglobulin in CMV-infected fetuses compared to healthy controls, as well as in symptomatic cCMV compared to asymptomatic ones. Differentiation between symptomatic and asymptomatic CMV fetuses: β2 microglobulin (AUC 0.98).
Hui (2022) [28]	Immune-related genes	Upregulation of genes related to innate immunity, IFN signaling, and immune response to viruses. Concurrent downregulation of neurodevelopmental genes.
Pedron (2007) [37]	CD8^+^ T cells	Higher levels of circulating activated, memory, and effector memory CD8^+^ T cell subsets.
Rizzo (2016) [39]	sHLA-G, β2 microglobulin	Higher β2 microglobulin level and sHLA-G levels and index in symptomatic CMV fetuses compared to asymptomatic and CMV-negative ones. Presence of free heavy chain in symptomatic fetuses.
Romanelli (2008) [40]	CMV-specific IgM	No differences between symptomatic and asymptomatic CMV fetuses.
Vermijlen (2010) [47]	γδ T cells	Presence of differentiated γδ T cells, expressing a CMV-specific TCR (detected at 21 weeks GA). Increased expression of NK receptor and cytotoxic mediator genes (granzyme A, perforin, etc.).
Vorontsov (2022) [48]	Immune-related proteins	1. Chemerin: significantly higher levels in cCMV infection, distinguishing severely symptomatic from asymptomatic fetuses. Lower levels in isolated SNHL fetuses compared to severely symptomatic ones. 2. Gal-3BP: Similar discriminative pattern as chemerin in terms of severity of clinical picture.

**Table 2:** Main findings of studies related to neonatal immune response to cCMV regarding innate immune components.

Author (Year)	Immune Component Studied	Results
Dantoft (2017) [23]	DCs	Upregulation of genes and gene networks related to IFN signaling, cytokine/chemokine signaling, and cell cycle. Downregulation of genes related to lipid metabolism and certain immunoregulatory functions.
Pighi (2024) [38]	NK cells	Predominance of mature NK cell subsets, with increased expression of NKG2C and reduced expression of NKG2A and NKG2D. Degranulating NK cells with higher expression of NKp46.
Semmes (2024) [43]	NK cells	Increased mature CD56^neg^CD16^+^ NK cell subset, with increased expression of activation and differentiation markers NKG2C and CD57. Increased transcriptional expression of innate immune genes and LAG-3.
Szala (2011) [45]	Complement	No differences in frequencies of MBL2-deficient genotypes or polymorphisms between cCMV-infected and CMV-negative neonates.
Vaaben (2022) [46]	NK cells	Presence of mature, differentiated, functional, and cytotoxic NK cell subsets (expressing granzyme B, perforin, and granulysin) in cCMV-infected neonates. Significantly higher frequency of the CD56^neg^ subset. Lower expression of the NKG2A receptor. Higher expression of the NKG2C receptor in symptomatic neonates.
Vermijlen (2010) [47]	γδ T cells	Higher percentages and absolute numbers of functional and oligoclonally expanded γδ T cells, which are activated, differentiated, express high levels of NK receptor, cytotoxic mediator, and chemokine/chemokine receptor genes, and secrete IFN-γ in response to CMV.
Yamaguchi (2023) [51]	Complement	Trend for higher levels of C3 in symptomatic cCMV-infected neonates compared to asymptomatic ones.

**Table 3:** Main findings of studies related to neonatal immune response to cCMV regarding adaptive immune components.

Author (Year)	Immune Component Studied	Results
Capretti (2020) [19]	CD8^+^ T cells	QuantiFERON^®^-CMV: All symptomatic neonates had a non-reactive or indeterminate result at birth. All neonates with LTI were symptomatic at birth and showed this pattern.
Chen (2015) [20]	CD4^+^ and CD8^+^ T cells	Gradual increase in CMV-specific CD4^+^ and CD8^+^ T cells over the first 3 years of life, with CD8^+^ T cells predominating and related to urine viral load reduction.
Elbou Ould (2004) [24]	CD4^+^ and CD8^+^ T cells	Preferential expansion of CD8^+^ over CD4^+^ T cells. Functional responses with activated and differentiated phenotypes, secreting IFN-γ upon CMV stimulation.
Gibson (2004) [26]	CD8^+^ T cells	Detectable CMV-specific CD8^+^ T cell responses in most cCMV infants, with stronger responses against IE-1. Their magnitude increased over time, correlating with decreasing viral load.
Gibson (2015) [27]	CD4^+^ and CD8^+^ T cells	No significant differences in memory CD4^+^ T cell frequencies or CD4^+^ and CD8^+^ T cell phenotypes between cCMV-infected and uninfected infants.
Huygens (2015) [29]	CD4^+^ and CD8^+^ T cells	Oligoclonal expansion of both subsets, but functional cytokine responses only in the CD8^+^ subset. Effector and late differentiation phenotypes. Increased PD-1 expression linked to T cell exhaustion, reversible by PD-1 blockade.
Lidehall (2013) [32]	CD4^+^ and CD8^+^ T cells	cCMV-infected infants secreted IFN-γ. Undetectable CD4^+^ T cell responses over the first 2 years of life.
Marchant (2003) [34]	CD8^+^ T cells	Oligoclonal expansion of CD8^+^ T cells, with activated and late differentiation phenotypes and functional responses (IFN-γ, MIP-1β/CCL4, and TNF-α secretion).
Medoro (2024) [35]	CD4^+^ and CD8^+^ T cells	Low CMV pp-65-specific CD4^+^ and CD8^+^ T cell responses over the first year of life, with higher frequencies of CD4^+^ CD28^−^ T cells and terminally differentiated, inhibitory and effector memory CD8^+^ T cells, compared to healthy controls. Similar profile of CD8^+^ T cell populations in cCMV infants with normal neurodevelopment. Higher frequencies of CD57^+^ PD-1^+^ CD8^+^ T cells found in asymptomatic cCMV infants and those with isolated SNHL. Higher frequencies of PD-1^+^ CD8^+^ T cells in cCMV infants with progressive SNHL.
Romanelli (2008) [40]	IgM antibody	Higher CMV-specific IgM in symptomatic cCMV.
Semmes (2024) [43]	CD4^+^ and CD8^+^ T cells	Increased activated, differentiated, and exhausted CD4^+^ T cells, with increased expression of cytotoxic mediator genes. Increased total and central memory/effector memory CD8^+^ T cell subsets. Expansion of NK-like CD8^+^ T cell populations, with increased expression of NK genes, which are cytotoxic and secrete IFN-γ in a FcγRIII-dependent way, thus mediating Antibody-Dependent Cellular Cytotoxicity.
Soriano-Ramos (2024) [44]	CD4^+^ and CD8^+^ T cells	Significantly lower T lymphocyte counts in cCMV-infected infants with LTI, however in a subgroup analysis for maternal infection <14 weeks GA, no differences were detected. No differences in CD4^+^ or CD8^+^ T cell counts, CD4^+^/CD8^+^ ratio, or CMV-specific IFN-γ CD4^+^ and CD8^+^ T cell responses with regard to symptoms at birth, LTI, or infants born postmaternal infection <14 weeks GA and LTI.

**Table 4:** Main findings of studies related to neonatal immune response to cCMV regarding immune-related markers that cannot be classified otherwise.

Author (Year)	Immune Component Studied	Results
Chen (2018) [21]	IL-33, sST2	Higher levels of IL-33 and its soluble receptor sST2 in cCMV-infected neonates.
Czech-Kowalska (2021) [22]	SNPs	SNP of IL-28 related to increased risk of cystic CNS lesions and ventriculomegaly. SNP of IL-1β related to reduced risk of ventriculomegaly.
Dantoft (2017) [23]	Immune cell markers and gene networks	Higher expression of NK and DC markers. Upregulation of IFN-induced, cell cycle, and erythrocyte gene networks in a cCMV-infected neonate.
Jedlinska-Pijanowska (2020) [30]	SNPs	Association of IL-1, IL-12, IL-28, and TLR4 SNPs with specific symptoms (reduced risk of splenomegaly, prematurity, higher risk of thrombocytopenia, hepatitis). SNP of CCL2/MCP-1: not associated with SNHL.
Kasztelewicz (2017) [31]	SNPs	Two CCL2/MCP-1 SNPs related to SNHL at birth; one also related to delayed-onset SNHL.
Liu (2007) [33]	Cytokines, chemokines	Increased levels of β-defensin 8 and macrophage-derived chemokine in cCMV-infected neonates. PF-4 and IL-25 increased in those with hepatitis.
Ouellette (2020) [36]	Immune gene signatures	Gene expression biosignatures successfully distinguished cCMV-infected from uninfected neonates, but not symptomatic from asymptomatic cases. A 16-gene classifier biosignature predicted the risk of late-onset SNHL.
Rovito (2017) [41]	TREC, KREC	1. TREC level: lower in cCMV-infected neonates. 2. KREC number and percentage: lower in cCMV-infected infants without LTI and correlated with viral load.
Rovito (2018) [42]	Immune-related genes	Upregulation of genes related to T-cell exhaustion (differentiation, effector, and inhibitory markers) in cCMV-infected neonates, especially in those with higher viral loads. Increased expression of anti-inflammatory gene pathways in cCMV-infected infants without LTI.
Wang (2021) [49]	Cytokines	Higher levels of Acrp30 and MMP-3 and lower levels of IL-1α in the CSF of CMV-infected neonates. Among cCMV neonates, higher MMP-3 levels related to severe neuroimaging abnormalities.
Wujcjicka (2017) [50]	SNPs	SNPs of IL-1α and IL-1β related to increased risk of cCMV infection and symptomatic cCMV. Co-existence of 5 SNPs (IL-1α, IL-1β, IL-6, IL-12β, TNF-α) related to increased risk of cCMV infection.
Yamaguchi (2023) [51]	Immune-related proteins	Eighty differentially excreted proteins in symptomatic cCMV/isolated SNHL, with top pathways identified being complement and coagulation cascade, platelet degranulation, and inflammation. Thirty-one differentially excreted proteins in cCMV infants with neuroimaging abnormalities, with top pathways identified being regulation of insulin-like growth factor transport and uptake and response to wounding.

## Data Availability

The original contributions presented in this study are included in the article/Supplementary Material. Further inquiries can be directed to the corresponding author.

## Supplementary Materials

The following supporting information can be downloaded at: https://www.mdpi.com/article/10.3390/v18020242/s1, Table S1: Main characteristics of included studies; Table S2: Outcome of included studies with regard to fetal immune response to cCMV; Table S3: Outcome of included studies with regard to neonatal immune response to cCMV; Table S4: Quality assessment of included cohort studies using the Joanna Brigg’s Institute Critical Appraisal tool; Table S5: Quality assessment of included case–control studies using the Joanna Brigg’s Institute Critical Appraisal tool; Table S6: Quality assessment of included cross-sectional studies using the Joanna Brigg’s Institute Critical Appraisal tool.

## Abbreviations

The following abbreviations are used in this manuscript:

cCMV	congenital Cytomegalovirus
SNHL	Sensorineural Hearing Loss
NDI	Neurodevelopmental Impairment
LTI	Long-Term Impairment
AF	Amniotic Fluid
SNPs	Single Nucleotide Polymorphisms
CD	Cluster of Differentiation
HCMV	Human Cytomegalovirus
DNA	Deoxyribonucleic Acid
PCR	Polymerase Chain Reaction
DBS	Dried Blood Spot
IE	Immediate-Early
EV	Extracellular Vesicles
RNA	Ribonucleic Acid
IP-10	Interferon-gamma-induced Protein-10
ITAC	Interferon-inducible T-cell Alpha Chemoattractant
IL	Interleukin
TRAIL	TNF-Related Apoptosis-Inducing Ligand
CRP	C-Reactive Protein
MIG	Monokine Induced by Gamma-interferon
RANTES	Regulated upon Activation, Normal T-cell Expressed and Secreted
IFN	Interferon
GAL-3BP	Galectin-3 Binding Protein
AUC	Area Under the Curve
HLA	Human Leukocyte Antigen
NK	Natural Killer
TCR	T-Cell Receptor
PMA	Phorbol Myristate Acetate
IgM	Immunoglobulin M
KIR	Killer Immunoglobulin Receptors
MIP	Macrophage Inflammatory Protein
CCL	Chemokine (C-C motif) Ligand
CCR	C-C motif Chemokine Receptor
CXCR	C-X-C motif Chemokine receptor
CXCL	Chemokine (C-X-C motif) Ligand
PD-1	Programmed cell Death protein-1
DCs	Dendritic Cells
MCP	Monocyte Chemoattractant Protein
TLR	Toll-Like Receptor
MBL	Mannose-Binding Lectin
C3	Complement component 3
TNF	Tumor Necrosis Factor
ADCC	Antibody Dependent Cellular Cytotoxicity
TREC	T-cell Receptor Excision Circles
KREC	Kappa-deleting Recombination Excision Circles
sST2	Soluble Suppressor of Tumorigenicity 2
PF-4	Platelet Factor 4
CSF	Cerebrospinal Fluid
Acrp30	Adipocyte-secreted Protein 30
MMP-3	Matrix-Metalloproteinase 3
ELISA	Enzyme-Linked Immunosorbent Assay
IGF	Insulin-like Growth Factor
CYP	Cytochrome
DEG	Differentially Expressed Genes
T-bet	T-box expressed in T-cells
LAG3	Lymphocyte-Activation Gene 3
APCs	Antigen-Presenting Cells
Th1	T-helper 1
NF-κB	Nuclear Factor kappa-light-chain-enhancer of activated B cells
MAPK	Mitogen-Activated Protein Kinase
MHC	Major Histocompatibility Complex
STAT	Signal Transducer and Activator of Transcription
UL	Unique Long
SGA	Small for Gestational Age
GA	Gestational Age
US	Ultrasound
MRI	Magnetic Resonance Imaging
ABR	Auditory Brainstem Response
CNS	Central Nervous System
