# Supplementary material for: Fetal and Neonatal Immune Response to Congenital Cytomegalovirus (cCMV) Infection: A Systematically Conducted Scoping Review
Source: Viruses. 2026 Feb 14;18(2):242. doi: 10.3390/v18020242 (PMC12945067; doi:10.3390/v18020242)
Supplement: Supplementary file 1 [file viruses-18-00242-s001.zip › viruses-4135022-supplementary.pdf]

Fetal and neonatal immune response to congenital Cytomegalovirus (cCMV) infection: a systematically conducted scoping review

Supplementary Table S1: Main characteristics of included studies.
Supplementary Table S2: Outcome of included studies with regard to fetal immune response to cCMV.
Supplementary Table S3: Outcome of included studies with regard to neonatal immune response to cCMV.
Supplementary Table S4: Quality assessment of included cohort studies using the Joanna Brigg's Institute Critical Appraisal tool
Supplementary Table S5: Quality assessment of included case-control studies using the Joanna Brigg's Institute Critical Appraisal tool
Supplementary Table S6: Quality assessment of included cross-sectional studies using the Joanna Brigg's Institute Critical Appraisal tool

Supplementary Table S1: Main characteristics of included studies.

**Author (year)**	**Country**	**Study design**	**cCMV participants or incident cases (N, diagnosis method)**	**Control participants or non-incident cases (N, definition)**	**Matching criteria**	**Method**	**Outcome**
Bourgon (2022) (18)	France	Case-control	40, fetuses with maternal primary infection 2 months preconceptionally or in the first trimester of pregnancy.<br>1. Fetal diagnosis: amniocentesis at Gestational Age (GA) 17 weeks minimum and at least 8 weeks after maternal infection (median GA 22.1 weeks, range 20.6-24.9 weeks).<br>2. Neonatal diagnosis: CMV-DNA PCR in urine, blood and saliva.	40, non-CMV infected, euploid fetuses (median GA at amniocentesis 22.0 weeks, range 20.0-25.0 weeks)	GA in amniocentesis, fetal gender	ELISA	Cytokine levels in amniotic fluid (IL-1α, IL-1β, IL-2, IL-4, IL-6, IL-8, IL-10, IL-13, IL-15, IL-16, IL-18, IL-33, Calgranulin A, Calgranulin C, CRP, CXCL6, CXCL13, Eotaxin (CCL11), GM-CSF, CXCL1, HMGB1, IFN-β, IFN-γ, IP-10, ITAC, M-CSF, MCP-1/CCL2, MIF, CXCL9, MIP-1α/CCL3, MIP-1β/CCL4, MIP-3α/CCL20, RANTES/ CCL5), TGF-β,<br>TNF-α, TRAIL).
Capretti (2020) (19)	Italy	Prospective cohort	10, neonates with positive urine PCR-CMV during the first 2 weeks of life and symptoms at birth	20, neonates with positive urine PCR-CMV during the first 2 weeks of life and lack of symptoms at birth	N/A	QuantiFERON®-CMV	$CD8^{+}$-T cell response
Chen (2015) (20)	USA	Prospective cohort	51, neonates with positive urine shell vial culture within the first 3 weeks of life or positive CMV early antigen by saliva testing, who developed a T cell immune response.	0, neonates with positive urine shell vial culture within the first 3 weeks of life or positive CMV early antigen by saliva testing, who did not develop a T cell immune response.	N/A	Flow cytometry	T cell response<br>1. $CD8^{+}$ T cells<br>2. $CD4^{+}$ T cells<br>3. Correlation with urine viral load

Chen (2018) (21)	China	Case-control	20, neonates with positive PCR for CMV DNA in urine and/or blood, all symptomatic at birth.	16, CMV-negative neonates with neonatal jaundice.	Age, gender	ELISA	1. IL-33, sST2 levels<br>2. Correlation with laboratory findings
Czech-Kowalska (2021) (22)	Poland	Prospective cohort	92, neonates with positive PCR for CMV DNA in urine	Group 1: 50, neonates with positive PCR for CMV DNA in urine and cystic CNS lesions on cranial US<br>Group 2: 26, neonates with positive PCR for CMV DNA in urine and cystic CNS lesions on MRI<br>Group 3: 29, neonates with positive PCR for CMV DNA in urine and ventricular dilatation on cranial US<br>Group 4: 43, neonates with positive PCR for CMV DNA in urine and ventricular dilatation on MRI	N/A	SNPs Genotyping - PCR	Association of 8 SNPs (cytokine and TLRs) with neuroimaging findings<br>1. IL1B rs16944<br>2. IL12B rs3212227<br>3. IL28B rs12979860<br>4. CCL2rs1024611<br>5. DC-SIGN rs735240<br>6. TLR2 rs5743708<br>7. TLR4 rs4986791<br>8. TLR9 rs352140
Dantoft (2017) (23)	UK	Case-control	1, symptomatic cCMV female infant born at 37 weeks GA	13, neonatal sepsis cases with positive blood culture for staphylococcus, born at 27-39 weeks GA (5 female, 8 male)	N/A	Whole blood transcriptomics – microarrays	1. Distribution of immune cell markers<br>2. Gene networks with differential expression
		Case-control	3, umbilical cord blood dendritic cells infected with HCMV	3, umbilical cord blood dendritic cells mock-infected	N/A	Transcriptomics	Monocyte-derived DCs:<br>1. Individual genes with differential expression at 6 and 16 hours post infection<br>2. Gene networks with differential expression at 6 and 16 hours post infection
Elbou Ould (2004) (24)	France	Case-control	15, fetuses (n=11) with CMV isolation on MRC5 monolayers[1] and positive CMV-DNA PCR in amniotic fluid prenatally (mean GA 29 weeks, range 22-38 weeks) and neonates (n=4) with	Group 1: 11, neonates from full-term uncomplicated pregnancies (GA range 38-40 weeks)	GA	Flow cytometry	T-cell response<br>1. Timing<br>2. Frequencies

[1] MRC5: Human Fetal Lung Fibroblast Cells monolayers. A protocol for isolating CMV from this cell line exists and was used in this study to diagnose congenital CMV infection.

			positive CMV-IgM and/or CMV isolation on MRC5 monolayer[2] at birth.	Group 2: 43, fetuses from pregnancies electively terminated due to fetal malformation unrelated to immune system development (GA range 17-38 weeks)			3. Phenotype<br>4. Function
Fabbri (2011) (25)	Italy	Case-control	47, CMV-infected fetuses diagnosed by positive viral culture and CMV DNA PCR in AF (median GA 22 weeks)	29, CMV-negative fetuses diagnosed by positive viral culture and CMV DNA PCR in AF (median GA 22 weeks)	N/A	Flow cytometry, ELISA	1. Total lymphocytes<br>2. $CD3^+$ $HLA\text{-}DR^+$ cells<br>3. CD4/CD8 T cell ratio<br>4. β2 microglobulin
		Nested prospective cohort	16, symptomatic cCMV, defined as histopathological findings of disseminated infection in terminated pregnancies or death in utero, abnormal ultrasound or MRI findings or clinical symptoms up to the age of 6 months	31, asymptomatic cCMV defined as pregnancies carried to term with lack of abnormal ultrasound or MRI findings or clinical symptoms up to the age of 6 months	N/A	Flow cytometry, ELISA	1. Total lymphocytes<br>2. $CD3^+$ $HLA\text{-}DR^+$ cells<br>3. CD4/CD8 T cell ratio<br>4. β2 microglobulin<br>5. CMV-specific IgM antibody
Gibson (2004) (26)	Italy, USA	Case-control	6, cCMV neonates diagnosed by detection of HCMV DNA in peripheral blood or isolation of the virus from the urine during the first 3 weeks of life, of whom<br>• N=5 born to HIV-1 seronegative women<br>• N=1 born to an HIV-1 seropositive woman, but infant HIV-negative	6, neonates HIV- and HCMV-negative	Age	ELISpot	CMV-specific $CD8^+$ T cell responses
		Nested prospective cohort	5, cCMV neonates diagnosed by detection of HCMV DNA in peripheral blood or isolation of the virus from the urine during the first 3 weeks of life, with detectable CMV-specific $CD8^+$ T cell responses	1, cCMV neonate diagnosed by detection of HCMV DNA in peripheral blood or isolation of the virus from the urine during the first 3 weeks of life, with undetectable CMV-specific $CD8^+$ T cell response	N/A	ELISpot	CMV-specific $CD8^+$ T cell responses<br>1. Timing of detection<br>2. pp65 and IE-1 response magnitude<br>3. Longitudinal responses<br>4. Relation to viral load

Gibson (2015) (27)	Italy, USA	Case-control	10, neonates with positive CMV-DNA in blood and/or virus isolation from urine during the first 3 weeks of life.	10, healthy CMV-negative infants and HIV-negative infants born to HIV-1-infected women	N/A	Flow cytometry	T cell response<br>1. Frequency<br>2. Phenotype
Hui (2022) (28)	Australia, Belgium	Case-control	13, fetuses with positive CMV-DNA PCR in AF of women who had serologic evidence of maternal primary infection in early pregnancy and/or fetal ultrasound abnormalities suggestive of CMV infection. Excluded if they received prenatal therapy with CMV hyperimmune globulin or antivirals (18-22 weeks GA).	13, euploid, structurally normal fetuses with negative CMV-DNA PCR in AF, from mothers with positive CMV serology in early pregnancy (18-22 weeks GA).	GA, fetal sex<br>Recruitment site (except 1 sample)	RNA sequencing	DEGs<br>1. Upregulated<br>2. Downregulated
Huygens (2015) (29)	Belgium	Case-control	36, fetuses and neonates with positive CMV-DNA PCR or CMV virus isolation by culture in AF or in newborn urine specimens collected during the 1st week of life	5, CMV-negative newborns	N/A	Flow cytometry, ELISA	T cell response<br>1.Repertoire<br>2.Phenotype
		Cross-sectional	36, fetuses and neonates with positive CMV-DNA PCR or CMV virus isolation by culture in AF or in newborn urine specimens collected during the 1st week of life	N/A	N/A	Flow cytometry, ELISA	T cell response<br>1. Function<br>2. Polyfunctionality<br>3. PD-1 expression<br>4. Blockade of PD-1
Jedlinska-Pijanowska (2020) (30)	Poland	Case-control	92, Caucasian neonates, positive CMV-DNA PCR in urine in the 21 first days of life. Excluded if multiple congenital anomalies, severe course of bacterial septic disease, or other TORCH infections.	141, Caucasian neonates, negative CMV-DNA PCR in urine. Excluded if multiple congenital anomalies, severe course of bacterial septic disease or other TORCH infections.	N/A	SNPs Genotyping – PCR	SNPs association with<br>1. cCMV disease<br>2. cCMV symptoms<br>3. Specific cCMV symptoms
Kasztelewicz (2017) (31)	Poland	Case-control	72, Caucasian neonates, positive CMV-DNA PCR in urine samples collected during the first 2–3 weeks of life.	398, Caucasian neonates, CMV-negative. Children with probable postnatal infection (i.e., diagnosed after	N/A	SNPs Genotyping – PCR	SNPs association with cCMV infection

				the third week of life), genetic disorders or congenital infections not related to CMV were excluded.			
		Case-control	22, cCMV neonates who failed Auditory Brainstem Response (ABR) at birth	34, cCMV neonates who passed ABR at birth	N/A	SNPs genotyping – PCR	SNPs association with SNHL at birth and at 6 months of age
Lidehall (2013) (32)	Sweden	Case-control	24, neonates with positive CMV-DNA in plasma, urine, leukocytes or dried blood spots during the first 3 weeks of life.	Group 1: 19, infants and children with postnatal CMV diagnosed by detectable CMV-IgG in plasma. If age < 12 months, positive CMV-DNA in urine was also required.<br><br>Group 2: 21, children admitted to the hospital for elective surgery, gastroscopy, cystoscopy or follow-up due to premature birth with negative CMV-IgG and CMV-PCR.	N/A	Flow cytometry, intracellular IFN-γ staining	T cell response<br><br>1. $CD8^+$ T cells<br>2. $CD4^+$ T cells
Liu (2007) (33)	China	Case-control	30, neonates with positive IE-CMV gene in urine, with hepatitis defined as clinical symptoms (jaundice, hepatosplenomegaly) and/or transaminasemia	Group 1: 30, neonates with positive IE-CMV gene in urine, without hepatitis symptoms or laboratory findings<br><br>Group 2: 30, CMV-negative infants with symptoms (jaundice, hepatosplenomegaly) and/or transaminasemia, consistent with hepatitis<br><br>Group 3: 12, CMV-negative infants, without symptoms or laboratory findings of hepatitis	N/A	Proteomics (SELDI-TOF Mass Spectrometry)	Serum proteins with differential expression in<br><br>1. cCMV with hepatitis<br>2. cCMV<br>3. cCMV without hepatitis<br>4. Hepatitis
Marchant (2003) (34)	Gambia, UK	Case-control	8, neonates with positive CMV-DNA PCR in urine during the first week of life.	15, neonates with negative CMV-DNA PCR in urine.	N/A	Flow cytometry, spectratyping, intracellular staining, ELISpot assay	$CD8^+$ T cell response<br><br>1. Proportion of dividing cells<br>2. Repertoire<br>3. Phenotype

							4. Function
Medoro (2024) (35)	USA	Case-control	21, infants aged ≤ 60 days with positive saliva or urine PCR for CMV during the first 3 weeks of life, confirmed by a second CMV PCR test (urine, blood or DBS)	5, CMV-uninfected infants aged ≤ 60 days in whom evaluation for active infection was negative	Age	Flow cytometry, intracellular staining	CMV pp65-specific $CD4^+$ and $CD8^+$ T cell cytokine responses (IFN-γ, IL-2, TNF- α, MIP-1β)
		Case-control	18, infants aged ≤ 60 days with positive saliva or urine PCR for CMV during the first 3 weeks of life, confirmed by a second CMV PCR test (urine, blood or DBS)	5, CMV-uninfected infants aged ≤ 60 days in whom evaluation for active infection was negative	Age	Flow cytometry	$CD4^+$ and $CD8^+$ T cell populations
		Case-control	12, symptomatic cCMV infants aged ≤ 60 days, diagnosed by a positive saliva or urine PCR for CMV during the first 3 weeks of life, and confirmed by a second CMV PCR test (urine, blood or DBS), including:<br>• N=7 with CNS symptoms<br>• N=5 without CNS symptoms (including N=2 with early onset hearing loss)	6, asymptomatic cCMV infants aged ≤ 60 days, diagnosed with a positive saliva or urine PCR for CMV during the first 3 weeks of life, and confirmed by a second CMV PCR test (urine, blood or DBS)	N/A	Flow cytometry	$CD8^+$ T cell populations
		Case-control	14, cCMV infants with normal neurodevelopment at 12 months of age	Group 1: 4, cCMV infants with developmental delay at 12 months, defined as score below the average range in at least one domain of Bayley III or IV or clinical diagnosis of developmental delay<br>Group 2: 5, CMV-uninfected infants aged ≤ 60 days in whom evaluation for active infection was negative, with normal neurodevelopment	N/A	Flow cytometry	$CD8^+$ T cell populations
		Case-control	9, cCMV infants with SNHL, defined as an audiologic threshold > 25 dB in either ear	14, infants with normal hearing, including<br>• N=9 cCMV infants<br>• N=5 CMV-negative infants	N/A	Flow cytometry	$CD8^+$ T cell populations

Ouellette (2020) (36)	USA	Case-control	80, neonates with positive culture or CMV-DNA PCR in urine (88%) and/or a positive CMV-DNA PCR from saliva (12%) during the 1st week of life. Positive saliva samples were all confirmed by CMV urine culture or PCR. Further divided into: ● Group 1: symptomatic, n=49<br>● Group 2: asymptomatic, n=31	10, CMV-negative healthy infants enrolled at well-child visits or prior to undergoing minor elective surgical procedures. Excluded if acute illness, exposure to antibiotics or steroids within 2 weeks of enrollment, or any underlying comorbidity.	Age, sex, GA, race	Whole blood transcriptomics – microarrays	1. Symptomatic cCMV biosignature<br>2. Asymptomatic cCMV biosignature<br>3. Biosignatures distinguishing between<br>● cCMV and controls<br>● Symptomatic and asymptomatic cCMV<br>4. Changes in biosignatures over time<br>5. Modular analysis of DEGs
Ouellette (2020) (36)	USA	Nested prospective cohort	24, cCMV infants who passed the initial newborn hearing screening and developed SNHL during the 3-year follow-up period	28, cCMV infants who passed the initial newborn hearing screening, had at least 900 days of follow-up and did not develop SNHL	N/A	Whole blood transcriptomics – microarrays	Classifier biosignature for late-onset SNHL
Pedron (2007) (37)	France	Case-control	16, CMV-positive fetuses diagnosed by a positive CMV DNA PCR in AF at 17-19 weeks GA, with samples collected between 22-34 weeks GA and at birth for one infant	Unknown number, full-term CMV-negative neonates sampled at birth	N/A	Flow cytometry	Circulating cytotoxic T lymphocytes ($CD8^+$ T cells)<br>1. Phenotype
Pighi (2024) (38)	Italy	Case-control	12, cCMV neonates diagnosed by a positive CMV DNA PCR in urine or blood during the first 3 weeks of life	5, healthy CMV-negative neonates born to mothers with CMV infection during pregnancy who did not transmit the infection to the fetus	N/A	Flow cytometry	NK cells<br>1. Phenotype<br>2. Degranulating cells phenotype
Rizzo (2016) (39)	Italy	Case-control	17, CMV-positive fetuses (N=15) and neonates (N=2) diagnosed by virus isolation and a positive CMV DNA PCR in AF (20-21 weeks GA) and/or urine in the first 2 weeks of life	39, CMV-negative fetuses, ascertained by lack of isolation of the virus and negative CMV DNA PCR in AF (20-21 weeks GA) and/or urine in the first 2 weeks of life	N/A	ELISA	sHLA-G<br>1. AF levels<br>2. Index<br>3. Free heavy chain<br>β2 microglobulin<br>1. AF levels

		Nested prospective cohort	12, symptomatic cCMV fetuses (N=11) and neonate (N=1), defined as the presence of ultrasound, immunopathological and histochemical findings in fetal organs and/or clinical and laboratory findings of cCMV	6, asymptomatic cCMV fetuses/ neonates, defined as the absence of clinical or laboratory findings of cCMV up to 6 years of age	N/A	ELISA	Soluble HLA-G<br>1. AF levels<br>2. Index<br>3. Free heavy chain<br>β2 microglobulin<br>1. AF levels
Romanelli (2008) (40)	France	Case-control	Symptomatic CMV-positive fetuses (N=5) and neonates (N=4), diagnosed by a positive CMV DNA PCR in AF after 19 weeks GA (median GA at amniocentesis 23 weeks) and signs of CMV disease at physical examination or ultrasound<br>Cord blood samples obtained at a median GA of 26 weeks and at birth.	8, asymptomatic CMV-positive fetuses and neonates, diagnosed by a positive CMV DNA PCR in AF after 19 weeks GA (median GA at amniocentesis 23 weeks), without signs of CMV disease at physical examination or ultrasound<br>Cord blood samples obtained at a median GA of 26 weeks and at birth.	N/A	ELISA	CMV-specific IgM levels
Rovito (2017) (41)	Netherlands	Case-control	99, children retrospectively diagnosed by positive CMV-DNA PCR in neonatal DBS at 5 years of age.	54, children with negative CMV-DNA PCR in neonatal DBS at 5 years of age.	Gender, month of birth, region	Real time quantitative PCR	TRECs and KRECs percentage, number and specific rearrangements in relation to cCMV<br>1. cCMV infection<br>2. Viral load<br>3. Symptoms at birth<br>4. LTI
Rovito (2018) (42)	Netherlands	Case-control	6, children with positive CMV-DNA PCR in neonatal DBS at 5 years of age and LTI at 6 years of age.	Group 1: 6, children with positive CMV-DNA PCR in neonatal DBS at 5 years of age, without LTI at 6 years of age<br>Group 2: 6, children with negative CMV-DNA PCR in neonatal DBS at 5 years of age.	Gender	Transcriptomics - next generation RNA sequencing	cCMV, LTI at 6 years of age and viral load in relation to expression of<br>1. Individual genes<br>2. Gene pathways<br>3. Selected genes related to T cell exhaustion
Semmes (2024) (43)	USA	Case-control	59, neonates with cCMV diagnosed by a positive CMV DNA PCR in cord blood	135, CMV-negative neonates from healthy, uncomplicated, term	Infant sex, race/ethnicity, maternal age (+/-	Flow cytometry	Immunophenotyping of<br>1. $CD4^+$ T cells

				pregnancies, ascertained by a negative CMV DNA PCR in cord blood	3 years), delivery year (+/- 3 years)		2. $CD8^+$ T cells<br><br>3. NK cells
		Case-control	13, neonates with cCMV diagnosed by a positive CMV DNA PCR in cord blood	12, CMV-negative neonates from healthy, uncomplicated, term pregnancies, ascertained by a negative CMV DNA PCR in cord blood	Infant sex, race/ethnicity, maternal age (+/- 3 years), delivery year (+/- 3 years)	RNA sequencing, flow cytometry	Transcriptional analysis and degranulation assays of<br><br>1. $CD8^+$ T cells<br><br>2. NK cells
Soriano-Ramos (2024) (44)	Spain	Case-control	42, symptomatic newborns with cCMV infection diagnosed by a positive CMV-DNA PCR in urine during the first 3 weeks of life	22, asymptomatic newborns with cCMV infection diagnosed by a positive CMV-DNA PCR in urine during the first 3 weeks of life	N/A	Flow cytometry	T cells<br><br>1. Total lymphocyte count<br><br>2. Total $CD4^+$ T cell count<br><br>3. Total $CD8^+$ T cell count<br><br>4. $CD4^+$/$CD8^+$ T cell ratio<br><br>5. CMV-specific IFN-γ secreting $CD4^+$ and $CD8^+$ T cell count
		Prospective cohort	18, cCMV newborns diagnosed by a positive CMV-DNA PCR in urine during the first 3 weeks of life, with long-term sequelae and at least 6 months of follow-up	46, cCMV newborns diagnosed by a positive CMV-DNA PCR in urine during the first 3 weeks of life, without long-term sequelae and at least 6 months of follow-up	N/A		
			4, newborns with cCMV infection diagnosed by a positive CMV-DNA PCR in urine during the first 3 weeks of life, whose mothers had a documented maternal primary infection < 14 weeks GA, with long-term sequelae and at least 6 months of follow-up	6, newborns with cCMV whose mothers had a documented maternal primary infection < 14 weeks GA, without long-term sequelae and at least 6 months of follow-up	N/A		T cells<br><br>1. Total lymphocyte count<br><br>2. CMV-specific IFN-γ $CD4^+$ and $CD8^+$ T cell responses
Szala (2011) (45)	Poland	Case-control	103, cCMV neonates diagnosed by symptoms, presence of CMV-IgM/IgA and/or positive CMV-DNA PCR during the first 3 weeks of life.	230, CMV-negative newborns from CMV-positive, non-transmitter mothers (n=59) or CMV-negative mothers (n=171)	N/A	Genotyping	1. Variant MBL2 alleles<br>2. MBL2 deficient genotypes
Vaaben (2022) (46)	Uganda	Case-control	16, neonates with positive CMV-DNA PCR in cord blood	69, neonates with negative CMV-DNA PCR in cord blood	N/A	Flow cytometry	NK cells<br><br>1. Frequency

							2. Phenotype<br>3. Function<br>4. Transcription factors<br>5. NK receptors
Vermijlen (2010) (47)	Belgium	Case-control	32 (13 fetuses, 20-29 weeks GA, and 19 newborns), positive CMV-DNA PCR and/or positive viral culture on amniotic fluid and/or on newborn urine collected during the first week of life.	34 (12 fetuses, 20-29 weeks GA, and 22 newborns), negative CMV-DNA PCR and/or positive viral culture on amniotic fluid and/or on newborn urine collected during the first week of life.	N/A	Flow cytometry, sequencing, spectratyping	γδ T cells:<br>1. Percentage and absolute number<br>2. Phenotype<br>3. Gene expression profiles<br>4. TCR repertoire<br>5. Function<br>6. Time of response
Vorontsov (2022) (48)	Israel	Case-control	14, cCMV fetuses, diagnosed by a positive CMV-DNA PCR in amniotic fluid samples (20-23 weeks GA)	10 CMV-negative fetuses, ascertained by a negative CMV-DNA PCR in amniotic fluid samples (20-23 weeks GA)		Proteomics (mass spectrometry proteomics)	Differentially excreted proteins
			6, severely symptomatic cCMV fetuses, diagnosed by a positive CMV-DNA PCR in amniotic fluid (20-23 weeks GA) and severe cerebral abnormalities in prenatal ultrasound and/or MRI (head circumference < -2SD of normal, ventriculomegaly, white matter abnormalities and cavitations, intracerebral hemorrhage, delayed cortical development)	8, asymptomatic cCMV fetuses, diagnosed by a positive CMV-DNA PCR in amniotic fluid (20-23 weeks GA) and normal clinical and laboratory parameters at birth and upon postnatal follow up of at least 12 months			
		Case-control	43, cCMV fetuses, diagnosed by a positive CMV-DNA PCR in amniotic fluid samples (20-23 weeks GA)	19, CMV-negative fetuses, ascertained by a negative CMV-DNA PCR in amniotic fluid samples (mean GA 21 weeks, range 20-23 weeks)		ELISA	Levels of<br>1.Chemerin<br>2.Gal-3BP
			17, severely symptomatic cCMV fetuses, diagnosed by a positive CMV-DNA PCR in amniotic fluid (mean GA 21 weeks, range 20-23 weeks) and severe cerebral abnormalities in prenatal ultrasound and/or MRI (head circumference < -2SD of normal, ventriculomegaly, white matter abnormalities and	26, asymptomatic cCMV fetuses, diagnosed by a positive CMV-DNA PCR in amniotic fluid (mean GA 21 weeks, range 20-23 weeks) and normal clinical and laboratory parameters at			

			cavitations, intracerebral hemorrhage, delayed cortical development)	birth and upon postnatal follow up of at least 12 months			
			6, cCMV fetuses diagnosed by a positive CMV-DNA PCR in amniotic fluid (20-23 weeks GA), with isolated SNHL diagnosed at birth or until the age of 24 months	Control group 1: 17, severely symptomatic cCMV fetuses, diagnosed by a positive CMV-DNA PCR in amniotic fluid (20-23 weeks GA) and severe cerebral abnormalities in prenatal ultrasound and/or MRI (head circumference < -2SD of normal, ventriculomegaly, white matter abnormalities and cavitations, intracerebral hemorrhage, delayed cortical development)<br><br>Control group 2: 26, asymptomatic cCMV fetuses, diagnosed by a positive CMV-DNA PCR in amniotic fluid (20-23 weeks GA) and normal clinical and laboratory parameters at birth and upon postnatal follow up of at least 12 months			
Wang (2021) (49)	China	Case-control	30, symptomatic cCMV neonates diagnosed by a positive CMV DNA PCR in urine and positive serum IgM and at least one clinical (hyperspasmia, physiological reflex, muscle tension changes) or imaging (intracranial calcification, ventricular dilatation, brain cyst, agyria) of the CNS.	30, CMV-negative neonates, ascertained by a negative CMV DNA PCR in urine and negative IgM in the serum, with fever and without CNS infection	N/A	Microarray, ELISA	Levels of 29 CNS-related cytokines (Acrp30, BDNF, b-NGF, CNTF, CRP, eotaxin, eotaxin-2, eotaxin-3, Fas, IL-4, GDNF, GM-CSF, IFN-γ, IL-10, IL-18, IL-1α, IL-1β, IL-6, IL-8, LIF, MCP-1, MIP-1β, MMP-2, MMP-3, TARC, TGF-β1, TIMP-1, TNF-α, VEGF) in<br><br>1. CSF<br><br>2. Serum

		Nested case-control	19, symptomatic cCMV neonates diagnosed by a positive CMV DNA PCR in urine and positive serum IgM, with severe imaging abnormalities of the CNS	11, symptomatic cCMV neonates diagnosed by a positive CMV DNA PCR in urine and positive serum IgM, with mild imaging abnormalities of the CNS	N/A	Microarray, ELISA	Levels of 3 CNS-related cytokines in CSF:<br>1. Acrp30<br>2. MMP-3<br>3. IL-1α
Wujcjicka (2017) (50)	Poland	Case-control	20, CMV-infected fetuses (N=7) and neonates (N=13), diagnosed by a positive PCR in AF, umbilical cord blood, urine or neonatal blood or plasma	31, CMV-uninfected neonates of women who were seronegative for CMV (IgM and IgG negative)	N/A	SNPs Genotyping - PCR	Association of 5 SNPs of cytokines (alone or in combination) with cCMV infection and symptomatic disease<br>1. IL1A _889 C > T<br>2. IL1B ώ3954 C > T<br>3. IL6 -174 G > C<br>4. IL12B _1188 A > C<br>5. TNF -308 G > A
Yamaguchi (2023) (51)	Japan	Case-control	9, cCMV infants, diagnosed by a positive CMV DNA PCR in urine or blood, of whom:<br>• N=5, symptomatic cCMV infants according to criteria by Rawlinson et al.<br>• N=4, infants with isolated SNHL	5, asymptomatic cCMV infants diagnosed by a positive CMV DNA PCR in urine or blood	N/A	Proteomics; Liquid Chromatography Mass Spectrometry (LC-MS), ELISA	1. Differentially Secreted Proteins<br>2. C3 levels
		Case-control	5, symptomatic cCMV infants, according to criteria by Rawlinson et al., diagnosed by a positive CMV DNA PCR in urine or blood, with neuroimaging abnormalities	9, cCMV infants diagnosed by a positive CMV DNA PCR in urine or blood, without neuroimaging abnormalities of whom:<br>• N=4, infants with isolated SNHL<br>• N=5, asymptomatic cCMV infants		Proteomics; Liquid Chromatography Mass Spectrometry (LC-MS), ELISA	1. Differentially Secreted Proteins<br>2. C3 levels

Supplementary Table S2: Outcome of included studies with regard to fetal immune response to cCMV.

**Author (year)**	**Component studied**	**Adjustment factors**	**Results**	**Limitations**
Bourgon<br><br>(2022) (18)	Cytokines<br><br>Innate and adaptive immunity	N/A	cCMV VS non-cCMV:<br><br>1. AF: increased IP-10/CXCL10, IL-18, ITAC/CXCL11, TRAIL<br><br>2. EV: increased IP-10<br><br>Symptomatic VS asymptomatic:<br><br>1. AF: increased IP-10/CXCL10, IL-18, TRAIL, CRP<br><br>2. EV: increased IP-10, IL-6, MCP-1, MIG, RANTES<br><br>Severe symptomatic VS non-severe symptomatic:<br><br>1. AF: increased IL-18, TRAIL, CRP<br><br>2. EV: increased TRAIL, MIG, RANTES	Low sensitivity of ELISA for EV cytokines
Elbou Ould<br><br>(2004) (24)	$CD8^+$ T cells<br><br>Adaptive immunity	N/A	cCMV VS controls:<br><br>1. Response detection: 22 weeks GA<br><br>2. Frequencies: preferential expansion of $CD8^+$ over $CD4^+$<br><br>3. Phenotype: switch to memory ($CD4^+$CD45RO+ and $CD8^+$ CD45RA-), activated ($CD3^+$/$CD8^+$/HLA-DR+) and terminally differentiated ($CD3^+$/$CD8^+$/CD28-)<br><br>4. Function: secretion of IFN-γ	N/A
Fabbri<br>(2011) (25)	T cells, CMV-specific IgM, β2 microglobulin<br><br>Adaptive immunity and immune markers	N/A	cCMV VS controls:<br><br>1. Total lymphocytes: increased proportion (median 86% (75-94%) versus 82% (68-93%), p=0.007)<br>2. $CD3^+$ HLA-DR^+ cells: increased proportion (median 5% (1-13%) versus 1% (1-5%), p=0.001)<br>3. CD4/CD8 ratio: decreased (median 1,6 (0,7-5,6) versus 2,9 (0,8-6,2), p=0.001)	

			4. β2 microglobulin: increased levels (median 10,4mg/L (1,8-22,4) versus 3,7 mg/L (3,1-4,8), p=0.001)<br><br>Symptomatic VS asymptomatic cCMV:<br><br>1. Total lymphocytes: increased proportion (p=0.005, cut-off 84,5%, AUC 0.8)<br>2. $CD3^+$ $HLA\text{-}DR^+$ cells: no statistically significant differences<br>3. CD4/CD8 ratio: no statistically significant differences<br>4. β2 microglobulin: increased levels (p<0.001, cut-off 11,5mg/L, AUC 0.98)<br>5. CMV-specific IgM ratio: increased levels (p<0.001, cut-off 3, AUC 0.95)<br>6. Best diagnostic accuracy: β2 microglobulin (comparable to combination of β2 microglobulin + CMV-specific IgM + CMV DNAemia +/- platelet count)	
Hui<br><br>(2022) (28)	Immune genes<br><br>Innate and adaptive immunity	Ultrasound abnormalities	cCMV VS controls:<br><br>1. Upregulated: IFN-signaling, immune response to viruses<br><br>2. Downregulated: neurodevelopmental<br><br>After exclusion of 3 pairs with ultrasound abnormalities:<br><br>1. Fewer DEGs<br><br>2. Upregulated: no difference in categories<br><br>3. Downregulated: only periaxin from the initial neurodevelopmental genes	1. Both maternal and fetal origin of AF cells<br><br>2. Small sample size<br><br>3. Absence of long-term follow-up<br><br>4. Low effective RNA library size<br><br>5. Some samples taken prior to 20 weeks GA (2/26)<br><br>6. Absence of validation with quantitative RT-PCR
Pedron<br>(2007) (37)	$CD8^+$ T cells<br><br>Adaptive immunity	N/A	cCMV VS controls:<br><br>1. Circulating $CD8^+$ T cells: higher levels of activated (HLA-DR+, p<0.001), effector memory (CD28-, p<0.0001) and memory ($CD18^{high}$, p=0.005) subsets	1. Fetuses sampled at different GA with a wide range<br>2. Unknown number of controls<br>3. Circulating and not CMV-specific CTLs<br>4. Small sample
Rizzo<br>(2016) (39)	sHLA-G, β2 microglobulin<br><br>Immune markers	N/A	cCMV VS controls:<br><br>1. sHLA-G<br>• AF levels: significantly higher in infected symptomatic fetuses than CMV-negative ones (median 73 ng/mL (69-79) versus 31ng/mL (29-40,2),	1. Small sample<br>2. Only one symptomatic cCMV infant born – did not investigate this biomarker in infants with various degrees of symptomatic cCMV disease

			p<0.001). The detection of a positive gradient in AF from symptomatic fetuses suggests fetal production.<br>• Index: higher in infected symptomatic fetuses compared to CMV-negative ones (19,5% versus 5,1%, p<0.001)<br>2. β2 microglobulin: significantly higher in infected symptomatic fetuses than CMV-negative ones (median 4,5 μg/mL versus 3,9μg/mL, p=0.042)<br><br>Symptomatic VS asymptomatic cCMV:<br><br>1. sHLA-G<br>• AF levels: significantly higher in symptomatic fetuses (median 73 ng/mL (69-79) versus 32ng/mL (28-42), p<0.001). The detection of a positive gradient in AF from symptomatic fetuses suggests fetal production. With a cut-off of 30ng/mL, AUC 0.86.<br>• Index: higher in symptomatic fetuses (19,5% versus 6%, p<0.001)<br>• Free heavy chain: tendency for its presence in symptomatic fetuses (p=0.074). Its presence has an AUC of 0.79 in differentiating between symptomatic and asymptomatic infection.<br>2. β2 microglobulin: significantly higher in symptomatic fetuses (median 4,5 μg/mL versus 3,6μg/mL, p=0.039)	3. Symptomatic disease at birth and long-term sequelae pooled together – biomarker not investigated in a cohort with asymptomatic infants presenting late-onset sequelae
Romanelli (2008) (40)	IgM antibody<br><br>Adaptive immunity	N/A	No statistically significant differences between symptomatic and asymptomatic CMV-positive fetuses.	4. Small sample<br>5. Symptomatic definition based only on clinical symptoms and ultrasound findings - outdated
Vermijlen<br><br>(2010) (47)	γδ T cells<br><br>Innate immunity	N/A	cCMV VS controls:<br><br>1. Response detection: 21 weeks GA<br><br>2. Phenotype: Differentiated (CD27-CD28-)<br><br>3. Increased expression of NK receptor genes, cytotoxic mediator genes (perforin, granzyme A)<br><br>4. Specific CDR3δ1 and CDR3γ8 sequences enriched	N/A
Vorontsov (2022) (48)	Immune-related proteins<br><br>Innate immunity	N/A	cCMV VS controls:<br><br>2. 59 differentially excreted proteins	1. Small sample<br><br>2. Inclusion of only severely symptomatic or totally

			3. Top pathways enriched: inflammatory proteins, cellular compromise and organismal injury and abnormalities<br>4. Significantly higher levels of chemerin in both symptomatic and asymptomatic cCMV fetuses (p=1.347e-05 and p=0.009 respectively)<br>Severely symptomatic VS asymptomatic cCMV:<br>1. 29 differentially expressed proteins<br>2. Top pathways enriched: inflammatory proteins, immunological disease, cellular compromise and organismal injury and abnormalities<br>3. Significantly higher levels of chemerin and GAL-3BP in severely symptomatic cCMV fetuses (p=1.347e-05 and p=0.000453 respectively)<br>4. Chemerin: 88.2% sensitivity, 100% specificity, AUC 0.98 for differentiation between the two groups<br>5. Gal-3BP: 88.2% sensitivity, 96.2% specificity, AUC 0.97 for differentiation between the two groups<br>SNHL VS severely symptomatic cCMV:<br>1. Significantly lower levels of chemerin and GAL-3BP in SNHL fetuses (p=1.518e-05 and p=0.002 respectively)<br>SNHL VS asymptomatic cCMV:<br>1. Not statistically significant differences in chemerin and GAL-3BP levels between groups (p=0.139 and p=0.672 respectively)	asymptomatic cases – cCMV has a spectrum of clinical manifestations and severity of disease
Wujcjicka (2017) (50)	SNPs of cytokines<br>Immune markers	N/A	cCMV infection:<br>1. CT haplotype (C allele of IL1A 889 C > T and T allele of IL1B 3954 C > T) associated with increased risk of cCMV infection SNP, increased the risk of congenital HCMV infection (2.6% vs 0%, OR $2.5x10^8$, p≤0.0001)<br>2. Multiple SNP analysis: variants CCGAG of IL1A, IL1B, IL6, IL12B and TNFA genes respectively, related to increased risk of cCMV infection (OR 7.94, 95%CI 1.38-45.69, p=0.026)	1. Small sample size<br>2. Combined analysis of fetuses and neonates

			Symptomatic cCMV disease:<br><br>1. CT haplotype (C allele of IL1A 889 C > T and T allele of IL1B 3954 C > T) associated with increased risk of symptomatic cCMV (4.9% vs 0%, OR 2.3x108, p≤0.0001)<br>2. CT heterozygotes in IL1A 889 C > T had increased risk of symptomatic cCMV (OR 2.86, 95% CI 0.24-33.90; p=0.045).	

Supplementary Table S3: Outcome of included studies with regard to neonatal immune response to cCMV.

**Author (year)**	**Component studied**	**Adjustment factors**	**Results**	**Limitations**
Capretti<br><br>(2020) (19)	CD8$^+$ T cells<br><br>Adaptive immunity	N/A	QuantiFERON®-CMV result:<br><br>1. T0: 16/30 reactive, all asymptomatic<br>2. T1: 17/29 reactive, all asymptomatic<br>3. All symptomatic neonates had a non-reactive or indeterminate result<br>4. Long-term follow up: 7/29 LTI, all symptomatic at birth, all with a non-reactive or indeterminate result<br>No correlation with median GA at maternal infection or urine viral load	1. Limitations of the testing method (sensitive to lymphopenia, detects only one cytokine (IFN-γ), uncommon HLA types not covered, qualitative result, IFN-γ threshold values not validated in neonates)<br><br>2. Lack of evaluation of CD4$^+$ T cell responses<br><br>3. Small sample size<br><br>4. Possible confounding by antiviral therapy<br><br>5. Lack of asymptomatic neonates with late-onset impairment
Chen<br><br>(2015) (20)	CD4$^+$ and CD8$^+$ T cells<br><br>Adaptive immunity	Age	cCMV, first 3 years of life:<br><br>1. Gradual increase of mean CMV specific CD4$^+$ and CD8$^+$ T cells<br>2. Urine viral load: decrease, related to CD8$^+$ T cell response<br>In each age group, the CD8$^+$ T cell response was higher than the group's CD4$^+$ T cell response.	1. Limited blood samples compared to urine ones<br><br>2. Small blood volumes<br><br>3. Small fraction of symptomatic infants in the cohort
Chen<br><br>(2018) (21)	Cytokines<br><br>Immune markers	N/A	1. Higher IL-33 and sST2 levels, correlated with infection status<br>2. Positive correlation between sST2 and ALT levels in both case and control groups	1. Only symptomatic at birth cCMV infants included in the cohort<br><br>2. Absence of follow-up for LTI<br><br>3. Non-specific infection markers

Czech-Kowalska (2021) (22)	SNPs of immune genes<br><br>Immune markers	N/A	2 SNPs associated with specific neuroimaging findings:<br><br>1. IL28B rs12979860 heterozygous (C/T) genotype and increased risk of cystic CNS lesions on cranial US (54% vs 26.2%, OR=3.31, 95%CI 1.37-8.01, p=0.0064) and in MRI (69.2% vs 31.1%, OR=4.97, 95%CI 1.84-13.43, p=0.001), and increased risk of ventriculomegaly in MRI (53.5% vs 31.8%, OR=2.46, 95%CI 1.03-5.90, p=0.04)<br>2. IL1B rs16944 heterozygous (G/A) or homozygous (A/A) and reduced risk of ventricular dilatation on cranial US (OR=0.38, 95%CI 0-0.93, p=0.034) and in MRI (OR=0.46, 95%CI 0.22-0.95, p=0.03)	1. Small sample size for detection of minor or infrequent alleles<br><br>2. Unknown HCMV strains – potential correlation of SNP results with specific strains<br><br>3. No adjustment for multiple comparisons – possible type 1 error
Dantoft (2017) (23)	Immune cell markers and immune gene networks<br><br>Immune markers	N/A	cCMV VS controls:<br><br>1. Elevated distribution levels in NK and dendritic cell markers. No differences in other immune cell populations.<br>2. Upregulation of erythrocyte network, cell-cycle network and IFN-induced gene subnetwork	Only one cCMV patient included.
	Monocyte-derived DCs<br><br>Innate immunity	N/A	Neonatal-infected VS mock-infected:<br><br>1. Upregulated genes: cytokine signaling, IFN-I response, IRF7, at 16 hrs TLR7, cell cycle<br>2. Downregulated genes: lipid and lipoprotein metabolism, genes with partial immunoregulatory roles<br>3. Upregulated networks: IFN, chemokine, cell cycle<br>4. Downregulated networks: GPCRs involved in complement and inflammation	
Elbou Ould (2004) (24)	$CD4^+$ and $CD8^+$ T cells<br><br>Adaptive immunity	N/A	cCMV:<br><br>1. Frequencies: preferential expansion of $CD8^+$ over $CD4^+$<br>2. Phenotype: switch to memory ($CD4^+$CD45RO+ and $CD8^+$ CD45RA-), activated ($CD3^+$/$CD8^+$/HLA-DR+) and terminally differentiated ($CD3^+$/$CD8^+$/CD28-)<br>3. Function: secretion of IFN-γ	N/A
Gibson (2004) (26)	$CD8^+$ T cells<br><br>Adaptive immunity	N/A	cCMV VS non-CMV infants:<br><br>1. Detectable CMV-specific $CD8^+$ T cell responses in 5/6 cCMV infants compared to 0/6 CMV-negative infants<br><br>cCMV infants with detectable response:<br><br>1. Timing of detection: 2/5 at Day 1 of age, 3/5 at 1-3 months of age<br>2. pp65 VS IE-1: 2/5 higher response to pp65, 3/5 higher response to IE-1	Small sample size

			3. Longitudinal responses: magnitude of response increased with age<br>4. Viral load: negative correlation; increasing CMV-specific CD8$^+$ T cell responses over time related to decreasing viral load.	
Gibson (2015) (27)	CD4$^+$ and CD8$^+$ T cells<br><br>Adaptive immunity	N/A	cCMV VS non-CMV infants:<br><br>1. Frequency: no differences in median frequencies of total memory CD4$^+$ T cells<br>2. Phenotype: no differences in memory CD4$^+$ (CD45RA$^-$ CCR7$^+$) and CD8$^+$ (CD45RA$^+$ CCR7$^-$) T cells.	Small sample size.
Huygens (2015) (29)	CD4$^+$ and CD8$^+$ T cells<br><br>Adaptive immunity	N/A	cCMV VS CMV-negative newborns:<br><br>1. Repertoire: oligoclonal expansion of both CD4$^+$ and CD8$^+$ T cells<br>2. Phenotype: high frequencies of late-differentiated CD27-CD28- T cells. Effector phenotype (Th1 CD4$^+$, Tc1 CD8$^+$), with decreased expression of CCR7, IL7R and increased expression of CD57, T-bet, CCR5.	N/A
		N/A	cCMV newborns' T cells:<br><br>1. Function: undetectable CD4$^+$ response, detectable CD8$^+$ response<br><br>2. Polyfunctionality: production of 1 or 2 cytokines, most commonly MIP-1β/CCL4<br><br>3. PD-1 expression: increased<br><br>4. Blockade of PD-1: upregulation of T cell responses after 7 days.	Lack of control group
Jedlinska-Pijanowska (2020) (30)	SNPs of immune genes<br><br>Immune markers	N/A	cCMV:<br><br>1. No association with cCMV disease<br>2. No association with symptomatic cCMV disease<br>3. 4 SNPs of IL-1, IL-12, IL-28 and TLR4 associated with particular symptoms<br>● IL1B rs16944 and reduced risk of splenomegaly<br>● IL12B rs3212227 and decreased risk of prematurity<br>● IL28B rs12979860 and increased risk of thrombocytopenia<br>● TLR4 rs4986791 and hepatitis<br>4. CCL2 rs1024611 was not related to SNHL at birth.	1. No correction for multiple testing-possible type I error<br>2. Small sample size<br>3. No adjustment for possible confounders made (e.g. different HCMV strains, primary or secondary maternal infection, time of the fetal infection)
Kasztelewicz (2017) (31)	SNPs of immune genes<br><br>Immune markers	Mother's CMV status, age at delivery	cCMV infection :<br><br>1. IL1B rs16944: higher frequency<br>2. TNF rs179996: higher frequency	1. Unknown GA at maternal infection<br>2. Unknown viral load in AF<br>3. No correction for multiple testing applied – possible type 1 error

		Symptoms at birth	SNHL at birth :<br><br>1. CCL2 rs13900: higher frequency<br>2. CCL2 rs1024611: higher frequency<br>SNHL at 6 months of age:<br><br>1. CCL2 rs13900: higher frequency<br>2. CCL2 rs1024611: no statistically significant differences detected	1. Only infants with early-onset SNHL included<br>2. No correction for multiple testing applied – possible type 1 error
Lidehall (2013) (32)	$CD4^+$ and $CD8^+$ T cells<br><br>Adaptive immunity	Age	cCMV VS CMV-negative: secretion of IFN-γ<br><br>cCMV VS postnatal CMV: no differences detected, impaired $CD4^+$ response persisted over the first 2 years of life.	N/A
Liu (2007) (33)	Cytokines, chemokines<br><br>Immune markers	N/A	1. cCMV VS CMV-negative: 4 proteins increased, among which β-defensin 8 and macrophage-derived chemokine<br>2. cCMV with hepatitis VS all control groups: 5 proteins increased, among which PF4/CXCL4 and IL-25<br>3. cCMV without hepatitis VS all other groups: 2 proteins increased, among which β-defensin 31<br>4. Hepatitis VS non-hepatitis: 4 proteins increased, among which pre-albumin, haptoglobin and human augmenter of liver regeneration.	N/A
Marchant (2003) (34)	$CD8^+$ T cells<br><br>Adaptive immunity	N/A	cCMV VS controls:<br><br>1. Proportion of dividing cells: no difference<br>2. Repertoire: oligoclonal expansion<br>3. Phenotype: activated, late differentiation (HLA-DR^+ $CD95^+$ $CD27^-$ $CD28^-$ $CD45RA^+$)<br>4. Function: cytotoxic (expressing granzyme A and high levels of perforin), producing cytokines (IFN-γ, MIP-1β/CCL4, TNF-α)	1. Only one symptomatic newborn included<br>2. Small sample size
Medoro (2024) (35)	$CD4^+$ and $CD8^+$ T cells<br><br>Adaptive immunity	N/A	cCMV VS controls:<br><br>1. CMV pp65-specific $CD4^+$ and $CD8^+$ T cells expressing IFN-γ, IL-2, TNF-α and MIP-1β: similar frequencies, which remained low during the first year of life, but were detected in 2/5 infants with an available sample during the second year of life<br>2. $CD8^+$ T cell populations: higher frequencies of terminally differentiated ($CD57^+$, $CD28^-$) and inhibitory (PD-1^+) $CD8^+$ T cells. Lower proportion of naïve ($CCR7^+$ $CD45RA^+$) $CD8^+$ T cells and higher proportion of effector memory ($CCR7^-$ $CD45RA^-$) and effector memory expressing RA ($CCR7^-$ $CD45RA^+$) subsets.	1. Most cCMV infants symptomatic<br>2. Responses only towards pp65 antigen were measured<br>3. Potential lower sensitivity of assays used compared to other available ones (eg ELISPOT)<br>4. Limited follow-up time<br>5. Small sample

			3. $CD4^+$ T cell populations: higher frequencies of $CD28^-$ $CD4^+$ T cells, no differences in CD57, PD-1 or memory subsets.<br><br>Symptomatic VS asymptomatic:<br><br>1. $CD8^+$ T cell populations: higher frequencies of $CD57^+$ $PD\text{-}1^+$ $CD8^+$ T cell subsets in asymptomatic infants and those with isolated SNHL.<br><br>Normal neurodevelopment VS developmental delay<br><br>1. T cell markers: higher frequencies of terminally differentiated ($CD57^+$, $CD28^-$) and inhibitory ($PD\text{-}1^+$) $CD8^+$ T cells in infants without developmental delay, which were eradicated during the first year of life.<br>2. Memory subsets: higher proportions of naïve ($CCR7^+$ $CD45RA^+$) $CD8^+$ T cells and lower proportion of effector memory ($CCR7^-$ $CD45RA^-$) and effector memory expressing RA ($CCR7^-$ $CD45RA^+$) subsets in infants with developmental delay and CMV-negative infants with normal neurodevelopment.<br><br>SNHL VS normal hearing:<br><br>1. Similar frequencies of $CD28^-$ $CD57^+$ $PD\text{-}1^+$ $CD8^+$ T cells<br>2. Infants with progressive SNHL: persistently elevated frequencies of $PD\text{-}1^+$ $CD8^+$ T cells	
Ouellette (2020) (36)	Immune gene signatures<br><br>Immune markers	N/A	cCMV VS controls:<br><br>1. Symptomatic cCMV biosignature: 2592 DEGs<br>2. Asymptomatic cCMV biosignature: 3324 DEGs<br>3. Biosignatures could distinguish between cCMV and healthy controls<br>4. Distinctive biosignatures persisted up to 3 years of age<br>5. Modular analysis:<br>● Birth: increased expression of modules related to interferon, T cells, B cells, NK cells and plasma cells, underexpression of modules related to monocytes and inflammation<br>● Year 1: normal expression of inflammation modules<br>● Year 2: overexpression of inflammation modules in asymptomatic group<br>● Year 3: overexpression of inflammation modules in both groups<br><br>Symptomatic VS asymptomatic:<br><br>1. Biosignatures could not distinguish between these groups	1. Small sample size<br>2. No correlations with viral load or functional immune assays were conducted<br>3. No proteomic validation of data<br>4. Possible bias due to development of SNHL in the asymptomatic group in rates higher than the ones reported in bibliography

			Late-onset SNHL VS no late-onset SNHL:<br><br>1. 16-gene signature related to late-onset SNHL detected, 97% AUC	
Pighi (2024) (38)	NK cells<br><br>Innate immunity	N/A	cCMV VS controls:<br><br>1. $CD56^{dim}CD16^{+}$ cells: significantly higher frequency of $CD57^{+}NKG2C^{+}$ (p = 0.0356) and memory-like populations (p = 0.0394), and a trend for higher expression of KIRs, CD57 and NKG2C, or a mature, terminally differentiated, or $KIR^{+}NKG2A^{-}CD57^{+}PD\text{-}1^{+}$ phenotype<br>2. Higher expression of NKG2C (geomean: 4223 vs 3337) and CD57 (geomean: 2141 vs 1311) on $CD56^{dim}CD16^{+}$, $CD56^{bright}CD16^{-}$ and $CD56^{low}CD16^{+}$ cells<br>3. $CD57^{-}NKG2C^{-}$ population: significant reduction (p = 0.0485)<br>4. Lower expression of NKG2A (geomean: 9502 vs 14306) and NKG2D (geomean: 10937 vs 12353)<br>5. Degranulating NK cells ($CD107a^{+}$): higher expression of NKp46 (geomean: 3386 vs 2308) and lower expression of NKG2C (geomean: 2431 vs 2829)<br>6. Typical populations of cCMV-infected newborns:<br>a. NK cells expressing high levels of NKG2C, CD57 and KIRs<br>b. Degranulating ($CD107a^{+}$) NK cells expressing high levels of NKG2C, CD57 and to a lesser extent NKp46.	1. Small sample<br>2. No comparisons in relation to symptoms<br>3. Control group being neonates of non-transmitting mothers and not of CMV-negative ones could have influenced results
Romanelli (2008) (40)	IgM antibody<br><br>Adaptive immunity	N/A	Statistically significant increased levels of CMV-specific IgM in symptomatic cCMV neonates VS asymptomatic ones (mean 46,4 +/- 25,4 g/dL VS 8,8 +/- 9,6 g/dL, p=0.03)	1. Small sample<br>2. Symptomatic definition based only on clinical symptoms and ultrasound findings - outdated
Rovito (2017) (41)	T and B cells<br><br>Immune markers	Prematurity and SGA for the TRECs	cCMV VS controls:<br><br>TRECs:<br><br>1. Percentage: lower, not statistically significant (p=0.073)<br>2. Number: lower (p=0.043)<br>3. Specific rearrangements: higher number of γδ T cells contained the Vδ1-Jδ1 rearrangement (p=0.019). No other statistically significant differences detected.<br>KRECs:<br><br>No differences detected.<br><br>High VS medium/low viral load:<br><br>TRECs:	1. Unknown trimester of maternal infection<br>2. Unknown maternal immune status to CMV (primary /non primary infection)

			1. Percentage and number: no difference<br>2. Specific rearrangements: higher percentage and number of cells containing Vδ1Jδ1 rearrangement ($p=0.022$). No other statistically significant differences detected.<br>KRECs:<br>1. Percentage: higher ($p=0.002$)<br>2. Number: higher ($p<0.001$)<br>3. Specific rearrangements: higher percentage and number of cells containing the cjintronRSS-Kde rearrangement.<br>Symptomatic VS asymptomatic: no differences detected.<br>LTI VS no LTI:<br>TRECs: no differences detected.<br>KRECs:<br>1. Percentage: lower<br>2. Number: lower<br>3. Specific rearrangements: no differences detected.	
Rovito (2018) (42)	Immune genes<br><br>Immune markers	N/A	cCMV VS controls:<br>1. Individual genes: no statistically significant differences<br>2. Pathways: no statistically significant differences<br>3. T cell exhaustion genes: increased expression of differentiation, effector and inhibitory markers<br>LTI VS non-LTI:<br>1. Individual genes: no statistically significant differences<br>2. Pathways: anti-inflammatory pathway associated with non-LTI. IL-4 gene drives a positive association with the absence of LTI.<br>3. T cell exhaustion genes: no statistically significant differences<br>Viral load:<br>1. Individual genes: no statistically significant differences detected<br>2. Pathways: innate immune response and NK-cell activation pathways associated with viral load, genes ISG15 and RSAD2 drive a positive association. Increased expression of differentiation (T-bet, CD57) and effector (granzyme, IFN-γ) markers, as well as inhibitory markers (PD-1 and LAG3)<br>3. T cell exhaustion genes: increased expression of differentiation, effector and inhibitory markers with higher viral load.	1. Lack of statistical power for individual genes<br>2. No standardized clinical-laboratory assessment at birth<br>3. 5/6 children with LTI at 6 years of age were symptomatic at birth<br>4. Unknown timing of fetal infection<br>5. Unknown maternal immune status to CMV (primary-non primary infection)<br>6. Small sample size<br>7. Possible degradation of archived DBS samples<br>8. Retrospective method of cCMV diagnosis might have led to misclassification

Semmes (2024) (43)	T and NK cells<br><br>Innate and adaptive immunity	N/A	cCMV VS controls:<br><br>1. $CD8^+$ T cells:<br>a. Immunophenotype: increased total and central memory/effector memory $CD8^+$ T cell subsets. Increased expression of differentiation marker CD57. Two clusters of $CD8^+$ T cells, one co-expressing NKG2A/NKG2C and one co-expressing FcγRIII/NKG2C expand significantly in cCMV neonates. Most of the FcγRIII $CD8^+$ T cell clusters canonical (αβ TCR) and Terminally Effector Memory expressing RA cells, with increased expression of CD57, PD-1 and NKG2C; 2 of the clusters non-canonical (γδ TCR).<br>b. Transcriptional analysis: 774 genes significantly upregulated (NK cell mediated immunity; p=0.0013, NK cell mediated cytotoxicity; p=0.0013, and regulation of NK cell immunity; p=0.0029) and 420 genes significantly downregulated. Increased expression (3- to 5-fold) of CCL3 (p=1.1 x 10^{-18}), CCL4 (p=2.1 x10^{-30}), CCL5 (p=1.7 x 10^{-20}), cytolytic molecules granzyme H (p=1.6 x10^{-11}), granzyme B (p= 3.8 x10^{-18}), perforin (p=9.2 x10^{-12}), granulysin (p= 2.7 x10^{-14}), and NKG7 (p=1.5 x10^{-24}). Increased expression of FcγRIIIa/CD16A genes (p=8.8 x10^{-26}), FcγRIIIb/CD16B genes (p=1.7 x10^{-13}) and KLRs. FcγRIII+ $CD8^+$ T cells had increased expression of KIRs, KLRs and other NK cell genes (CD244, NCR1/NKp46, NCAM1, TYROBP), as well as cytotoxic genes (granzyme B, granzyme H, perforin, granulysin and NKG7). FcγRIII – $CD8^+$ T cells had increased expression of markers of naïve T cells (CCR7 and IL-7R). Additionally, $FcγRIII^+$ $CD8^+$ T cells had increased expression of HHEX, IRF5, EOMES and T-bet transcription factors, with T-bet being the most upregulated, and decreased expression of MEOX1 and BCL11B.<br>c. Function: $FcγRIII^+$ $CD8^+$ T cells degranulate (CD107a) and produce IFN-γ in an antigen-specific, antibody-dependent manner, with enhanced degranulation after IL-15 pretreatment.<br>2. $CD4^+$ T cells:<br>a. Immunophenotype: lower proportion of total $CD4^+$ T cells, no differences in memory or Treg subsets, increased activated ($HLA\text{-}DR^+$), differentiated ($CD57^+$) and $PD\text{-}1^+$ $CD4^+$ T cells.<br>b. Transcriptional analysis: 25 genes significantly and 37 genes significantly downregulated. Increased expression (5- to 7-fold) of CCL5 (p=1.1 x10^{-8}), NKG7 (p=1.97 x10^{-6}) and granzyme H (p=5.8 x10^{-6}).<br>3. NK cells:	1. Only asymptomatic infants included<br>2. Long-term outcome data not available<br>3. Preterm or multiple gestation infants not included<br>4. Longitudinal analysis of phenotypes and transcriptional changes not performed<br>5. Transcriptional and functional analyses performed on a very small sample due to limited sample availability

			a. Immunophenotype: increased $CD56^{neg}CD16^{+}$ NK cells, several NK subsets activated and differentiated as displayed by increased expression of CD57, increased expression of NKG2C.<br>b. Transcriptional analysis: 29 genes significantly upregulated (innate immune response; p=0.0001, response to virus; p=0.00025, and type I IFN signaling; p=0.00067) and 12 genes significantly downregulated. Increased expression of LAG3 (4-fold, p=2.3 x10^{-10}) and JAK-MIP1 (5-fold, p=0.00253).<br>c. Function: similar degranulation and IFN-γ production after antibody stimulation.	
Soriano-Ramos (2024) (44)	T cells<br><br>Adaptive immunity	N/A	Symptomatic VS asymptomatic cCMV newborns<br><br>2. No statistically significant differences between groups in total lymphocyte count, $CD4^{+}$ T cell count, $CD8^{+}$ T cell count, $CD4^{+}/CD8^{+}$ T cell ratio or CMV-specific $CD4^{+}$ and $CD8^{+}$ T cell responses (p values not available)<br><br>cCMV with sequelae VS cCMV without sequelae:<br><br>3. Significantly lower T lymphocyte count in newborns with sequelae by multivariate analysis (aOR=0.549 per 1000 lymphocytes, 95% CI 0.323-0.833, p=0.012)<br>4. By univariate analysis, significantly lower $CD4^{+}$ T cell count (1454 vs 2143 $CD4^{+}$ T lymphocytes/mL, P = .02) and $CD4^{+}/CD8^{+}$ T cell ratio in newborns with sequelae (1.7 vs 2.4, p=0.03), but not by multivariate analysis<br>5. No statistically significant associations with CMV-specific IFN-γ responses of $CD4^{+}$ and $CD8^{+}$ T cells<br><br>Documented maternal primary infection <14 weeks GA<br><br>cCMV with sequelae VS cCMV without sequelae:<br><br>1. No statistically significant differences between total lymphocyte count or CMV-specific IFN-γ $CD4^{+}$ and $CD8^{+}$ responses	1. Definition of symptomatic infection includes newborns with isolated neuroimaging findings – possible overdiagnosis of symptomatic infection<br>2. Cranial Ultrasound categorized as abnormal when non-specific finding of Lenticulostriate Vasculopathy was present<br>3. House-made technique<br>4. Small sample of documented maternal primary infection <14 weeks GA (N=10)<br>5. No functional studies of T cells other than IFN-γ performed
Szala<br><br>(2011) (45)	Complement<br><br>Innate immunity	N/A	No differences detected between groups in frequencies of MBL2 deficient genotypes or MBL2 variant alleles.	N/A
Vaaben (2022) (46)	NK cells<br><br>Innate immunity	Malaria	cCMV VS negative controls:<br><br>1. Total frequencies: no difference (p=0.31)	1. Small sample size<br>2. Lack of long-term follow up<br>3. Only 2 symptoms at birth investigated (microcephaly, SGA)

			2. Phenotype: higher levels of mature/differentiated subsets and higher frequency of CD56- NK cells (p=0.02)<br>3. Function-Cytotoxicity: expression of at least one cytotoxic mediator; higher expression of granzyme B in all NK subsets (CD56- p<0.001; $CD56^{dim}$ p=0.002; $CD56^{bright}$ p=0.008), perforin in CD56- (p=0.003) and $CD56^{bright}$ (p=0.04) NK cells, and granulysin in $CD56^{bright}$ NK cells (p=0.01). Usually co-expression of mediators.<br>4. Transcription factors: no differences in expression of T-bet, eomes or Ki67<br>5. NK receptors: lower expression of NKG2A, higher expression of NKG2C only in symptomatic cCMV neonates (p=0.005).	4. Lack of adjustment for multiple comparisons
Vermijlen (2010) (47)	γδ T cells<br><br>Innate immunity	N/A	cCMV VS controls:<br><br>1. Higher percentage and absolute number<br>2. Phenotype: activated ($HLA\text{-}DR^{+}$) and differentiated ($CD27^{-}$ $CD28^{-}$)<br>3. Increased expression of NK receptor genes, cytotoxic mediator genes (granzyme A, B, perforin), chemokine and chemokine receptor genes (MIP-1α/CCL3, MIP-1β/CCL4, RANTES/CCL5, CCR5 ligands, and receptors CCR5 and CX3CR1), decreased CCR7 expression, increased IFN-γ gene expression and transcription factors associated with its production (T-bet, eomes)<br>4. Oligoclonal expansion: Public Vγ8Vδ1 TCR, germline encoded, consisting of restricted CDR3δ1 and CDR3γ8 repertoire, that recognizes CMV-infected cells<br>5. Function: IFN-γ production (in vitro stimulation and recognition by public TCR), killing of infected cells, restriction of viral replication in vitro	N/A
Wang (2021) (49)	CNS-related cytokines<br><br>Immune markers	N/A	cCMV VS controls:<br><br>1. Acrp30: significantly higher levels in CSF by microarray (66.036,29 +/- 8.383,33 vs 49.192,18 +/- 6.285,92, p<0.05) and ELISA (39,76 +/- 2,01 pg/mL vs 7,75 +/- 0,1 pg/mL, p<0.001)<br>2. MMP-3: significantly higher levels in CSF by microarray (995,65 +/- 189,78 vs 714,66 +/- 117,22, p<0.05) and ELISA (1,40 +/- 2,13 ng/mL vs 0,18 +/- 0,45 ng/mL, p=0.003)<br>3. IL-1α: significantly lower levels in CSF by microarray (672,62 +/- 24,28 vs 735,47 +/- 21,69, p<0.05) and ELISA (2,36 +/- 0,99 pg/mL vs 2,91 +/- 0,78 pg/mL, p=0.02)<br>4. No statistically significant differences in cytokine levels in the serum<br><br>cCMV with severe vs mild imaging abnormalities:<br><br>1. MMP-3: higher levels in CSF related to more severe imaging abnormalities (p=0.021)<br>2. No statistically significant differences in Acrp30 or IL-1α CSF levels	1. Control group consisting of neonates with fever and not healthy ones<br>2. CSF samples used, difficult and invasive to obtain

Wujcjicka (2017) (50)	SNPs of cytokines<br><br>Immune markers	N/A	cCMV infection:<br><br>1. IL1A rs1800587 and IL1B rs1143634 associated with increased risk of cCMV infection (2.6% vs 0%, OR $2.5x10^{8}$, p≤0.0001)<br>2. Multiple SNP analysis: coexistence of IL1A rs1800587, IL1B rs1143634, IL6 rs1800795, IL12B rs3212227 and TNFA rs1800629, related to increased risk of cCMV infection (OR 7.94, 95%CI 1.38-45.69, p=0.026)<br><br>Symptomatic cCMV disease:<br><br>1. IL1A rs1800587 and IL1B rs1143634 associated with increased risk of symptomatic cCMV (4.9% vs 0%, OR 2.3x108, p≤0.0001)<br>2. IL1A rs1800587 had increased risk of symptomatic cCMV (OR 2.86, 95% CI 0.24-33.90; p=0.045).	1. Small sample size<br>2. Combined analysis of fetuses and neonates
Yamaguchi (2023) (51)	Immune proteins, complement<br><br>Immune markers, innate immunity	N/A	Symptomatic cCMV and isolated SNHL VS asymptomatic:<br><br>1. 80 differentially excreted proteins; 65 upregulated and 11 downregulated >1.5 fold, top pathways: complement and coagulation cascade, platelet degranulation and inflammatory response<br>2. C3: higher levels, but not statistically significant (mean 58,8mg/dL vs 43,5mg/dL, p=0.07)<br><br>Neuroimaging abnormalities (symptomatic cCMV) VS normal neuroimaging (isolated SNHL/asymptomatic):<br><br>1. 31 differentially excreted proteins; 30 upregulated and 1 downregulated, top pathways: regulation of insulin-like growth factor transport and uptake by IGF proteins and response to wounding<br>2. C3: no statistically significant differences	1. Small sample size<br>2. No healthy control group included<br>3. Validation in a small number of proteins due to limited sample volume

Supplementary Table S4: Quality assessment of included cohort studies using the Joanna Brigg's Institute Critical Appraisal tool

Author (year)	1. Were the two groups similar and recruited from the same population?	2. Were the exposures measured similarly to assign people to both exposed and unexposed groups?	3. Was the exposure measured in a valid and reliable way?	4. Were confounding factors identified?	5. Were strategies to deal with confounding factors stated?	6. Were the groups/participants free of the outcome at the start of the study (or at the moment of exposure)?	7. Were the outcomes measured in a valid and reliable way?	8. Was the follow up time reported and sufficient to be long enough for outcomes to occur?	9. Was follow up complete, and if not, were the reasons to loss to follow up described and explored?	10. Were strategies to address incomplete follow up utilized?	11. Was appropriate statistical analysis used?	Total
Capretti (2020) (19)	Y	Y	Y	Y	Y	Y	Y	Y	Y	N	Y	10/11
Chen (2015) (20)	Y	Y	Y	Y	Y	N	Y	Y	Y	N	Y	9/11
Czech-Kowalska (2021) (22)	Y	Y	Y	N	N	Y	Y	Y	Y	Y	Y	9/11
Gibson (2004) (26)	Y	Y	Y	N	N	Y	Y	Y	Y	U	Y	8/11
Ouellette (2020) (36)	Y	Y	Y	Y	U	Y	Y	Y	Y	U	Y	9/11
Soriano-Ramos (2024) (44)	Y	Y	Y	Y	Y	Y	Y	Y	Y	U	Y	10/11

Supplementary Table S5: Quality assessment of included case-control studies using the Joanna Brigg's Institute Critical Appraisal tool

Author (year)	1. Were the groups comparable other than the presence of disease in cases or the absence of disease in controls?	2. Were cases and controls matched appropriately?	3. Were the same criteria used for identification of cases and controls?	4. Was exposure measured in a standard, valid and reliable way?	5. Was exposure measured in the same way for cases and controls?	6. Were confounding factors identified?	7. Were strategies to deal with confounding factors stated?	8. Were outcomes assessed in a standard, valid and reliable way for cases and controls?	9. Was the exposure period of interest long enough to be meaningful?	10. Was appropriate statistical analysis used?	Total
Bourgon (2022) (18)	Y	Y	Y	Y	Y	Y	Y	Y	Y	Y	10/10
Chen (2018) (21)	Y	Y	Y	Y	Y	Y	Y	Y	U	Y	9/10
Dantoft (2017) (23)	Y	Y	Y	Y	Y	N/A	N/A	Y	U	Y	7/10
Elbou Ould (2004) (24)	Y	Y	Y	Y	Y	U	U	Y	Y	Y	8/10
Fabbri (2011) (25)	U	U	Y	Y	Y	N	N	Y	Y	Y	6/10
Gibson (2004) (26)	U	U	U	Y	Y	N	N	Y	Y	Y	5/10
Gibson (2015) (27)	U	U	Y	Y	Y	U	N	Y	Y	Y	6/10
Hui (2022)	Y	Y	Y	Y	Y	Y	Y	Y	Y	Y	10/10

(28)											
Huygens (2015) (29)	U	U	Y	Y	Y	N	N	Y	Y	Y	6/10
Jedlinska-Pijanowska (2020) (30)	N	U	Y	Y	Y	Y	N	Y	Y	Y	7/10
Kasztelewicz (2017) (31)	N	U	Y	Y	Y	Y	N	Y	Y	N	6/10
Lidehall (2013) (32)	U	U	Y	Y	Y	Y	Y	Y	Y	Y	8/10
Liu (2007) (33)	U	U	Y	Y	Y	N	N	Y	Y	Y	6/10
Marchant (2003) (34)	U	U	Y	Y	Y	N	N	Y	Y	Y	6/10
Medoro (2024) (35)	Y	Y	Y	Y	Y	U	N	Y	Y	Y	8/10
Ouellette (2020) (36)	Y	Y	Y	Y	Y	Y	Y	Y	Y	Y	10/10
Pedron (2007) (37)	U	N	Y	Y	Y	N	N	Y	Y	Y	6/10
Pighi (2024) (38)	N	U	Y	Y	Y	U	N	Y	Y	Y	6/10
Rizzo (2016) (39)	U	U	Y	Y	Y	N	N	Y	Y	Y	6/10

Romanelli (2008) (40)	U	Y	Y	Y	Y	N	N	Y	Y	Y	7/10
Rovito (2017) (41)	U	Y	Y	Y	Y	Y	Y	Y	Y	Y	9/10
Rovito (2018) (42)	Y	Y	Y	Y	Y	Y	N	Y	Y	Y	9/10
Semmes (2024) (43)	Y	Y	Y	Y	Y	Y	N	Y	Y	Y	9/10
Soriano-Ramos (2024) (44)	U	U	Y	Y	Y	N	N	Y	Y	Y	6/10
Szala (2011) (45)	Y	Y	Y	Y	Y	N	N	Y	Y	Y	8/10
Vaaben (2022) (46)	Y	Y	Y	Y	Y	Y	Y	Y	Y	Y	10/10
Vermijlen (2010) (47)	U	N	Y	Y	Y	Y	N	Y	Y	Y	7/10
Vorontsov (2022) (48)	U	U	Y	Y	Y	Y	Y	Y	Y	Y	8/10
Wang (2021) (49)	Y	U	Y	Y	Y	N	N	Y	Y	Y	7/10
Wucjicka (2017) (50)	U	U	Y	Y	Y	N	N	Y	Y	Y	6/10
Yamaguchi	Y	Y	Y	Y	Y	N	N	Y	Y	Y	8/10

(2023) (51)										

Supplementary Table S6: Quality assessment of included cross-sectional studies using the Joanna Brigg's Institute Critical Appraisal tool

Author	1. Were the criteria for inclusion in the sample clearly defined?	2. Were the study subjects and the setting described in detail?	3. Was the exposure measured in a valid and reliable way?	4. Were objective, standard criteria used for measurement of the condition?	5. Were confounding factors identified?	6. Were strategies to deal with confounding factors stated?	7. Were the outcomes measured in a valid and reliable way?	8. Was appropriate statistical analysis used?	Total
Huygens (2015) (29)	Y	Y	Y	Y	N	N	Y	Y	6/8
